# Unlocking Li/Mg‐Based Photochemical Nitration Platforms for Anticancer Drug Discovery

**DOI:** 10.1002/advs.77873

**Published:** 2026-09-25

**Authors:** Qian Wan, Jiali Zhu, Shanshuo Liu, Luchuan Ou, Huizhen Wang, Yu Lan, Zhou Dong, Yihang Bai, Wei Yang, Shihan Liu, Yan Qiao, Shi‐Jun Li, Zhigang Ren, Linbin Niu

**Affiliations:** ^1^ College of Chemistry State Key Laboratory of Antiviral Drugs and Pingyuan Laboratory Zhengzhou University Zhengzhou Henan People's Republic of China; ^2^ Department of Infectious Diseases State Key Laboratory of Antiviral Drugs Pingyuan Laboratory the First Affiliated Hospital of Zhengzhou University Zhengzhou People's Republic of China; ^3^ Department of Pathophysiology School of Basic Medical Sciences Zhengzhou University Zhengzhou Henan People's Republic of China; ^4^ State Key Laboratory of Antiviral Drugs Henan Normal University Xinxiang Henan People's Republic of China; ^5^ School of Chemistry and Chemical Engineering Chongqing Key Laboratory of Chemical Theory and Mechanism Chongqing University Chongqing People's Republic of China; ^6^ Key Laboratory of Special Functional Molecular Materials (Zhengzhou University) Ministry of Education Zhengzhou Henan People's Republic of China; ^7^ College of Chemistry and Molecular Sciences Henan University Kaifeng Henan People's Republic of China

**Keywords:** anticancer drugs, Li/Mg‐based photochemistry, nitration, nitro radicals

## Abstract

The low toxicity and cost‐effectiveness of light main‐group metal photochemical systems render them highly suitable for drug development, while the redox inertness of light main‐group metals significantly hinders this goal. Herein, through the in situ assembly of readily available lithium nitrate, methoxyacetic acid, and aniline substrates, an unprecedented, mild lithium‐based photochemical system capable of producing valuable nitro radicals is established. This system enables selective C(sp^2^)–H (di‐)nitration of anilines for synthesizing nitro‐aromatic compounds as potential antitumor candidates. Further, replacing lithium nitrate with magnesium nitrate makes the reaction conditions milder, allowing lower‐energy green‐light irradiation for the (di‐)nitration. The two main‐group metal photochemical systems also enable direct late‐stage functionalization of complex drug molecules, eliminating the risk of transition metal residue and drastically simplifying the workup procedures of pharmaceutical manufacturing processes.

## Graphical abstract

Two low‐toxicity light main‐group metal photochemical systems based on lithium and magnesium are constructed for selective C(sp^2^)‐H (di)nitration of anilines. The obtained dinitrated products exhibit anti‐HCC (hepatocellular carcinoma) and anti‐KYSE30 cell activities, holding promise as novel antitumor candidates.

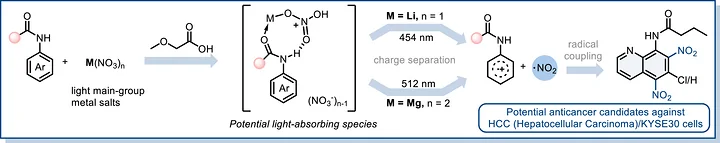

## 1 Introduction

**FIGURE 1:**
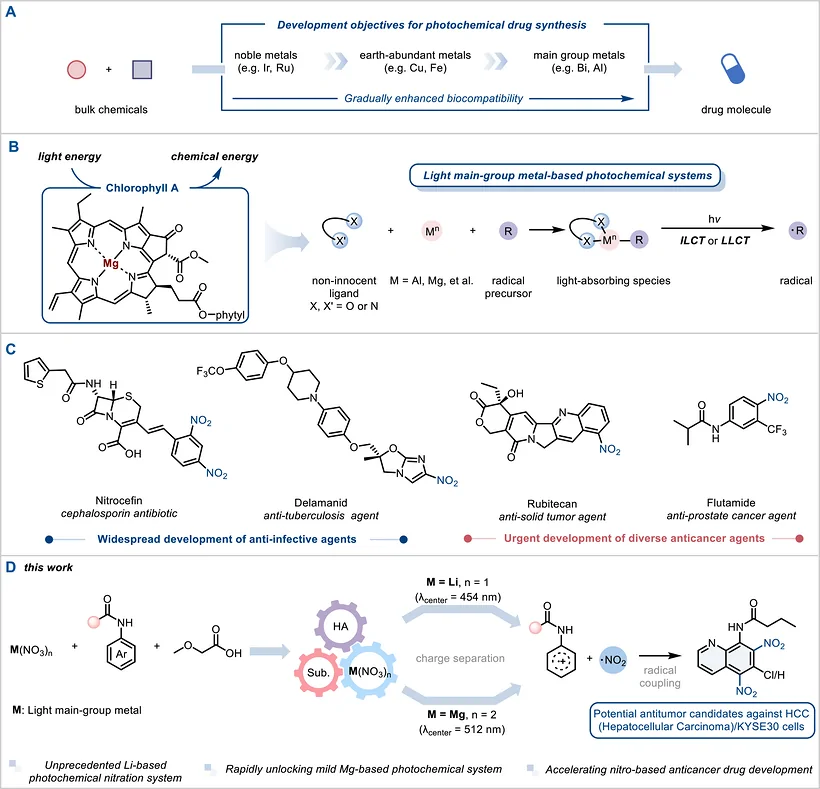
Main‐group metal‐based photochemical synthesis of drug molecules. (A) Metal‐based photochemical strategy for the preparation of drug molecules. (B) Natural and artificial light main‐group metal photochemical systems. (C) Representative nitro‐containing drug molecules. (D) This work: lithium or magnesium‐based photochemical systems for nitro‐based anticancer drug development.

In drug development, toxic metal residues represent a central concern for quality control and safety assessment [1, 2]; therefore, avoiding the introduction of toxic metals during drug molecule preparation is of great significance. Although tremendous efforts have been made in recent decades to replace toxic precious metals with less toxic 3d transition metals, these 3d transition metals may still fail to meet drug safety standards [3, 4]. In this regard, main‐group metals offer unique advantages due to their substantially high acceptable human exposure limits [5, 6]. Additionally, their great natural crustal abundance and low environmental toxicity greatly reduce the waste treatment costs associated with pharmaceutical manufacturing (Figure 1A) [7, 8]. Therefore, leveraging main‐group metals to access drug molecules is a highly advantageous strategy.

Using mild light energy to expand the application of main‐group metals in drug synthesis remains a challenging but promising goal. Since main‐group metals lack low‐energy d‐orbitals to accommodate photoexcited electrons or undergo redox changes, constructing mild main‐group photochemical systems typically requires the use of non‐innocent ligands [9, 10, 11, 12] (such as azaarenes or catecholates) to enable intraligand or ligand‐to‐ligand charge transfer, thereby generating reactive radical species [13, 14, 15]. This represents the fundamental guiding principle for developing main‐group photochemical systems [13, 14, 15, 16, 17, 18, 19, 20, 21, 22, 23, 24, 25, 26, 27, 28, 29, 30, 31, 32, 33, 34, 35, 36, 37, 38]. Nature provides an exemplary paradigm for the utilization of main‐group metal photochemistry, particularly in the context of more sustainable light main‐group metal photochemistry: magnesium‐containing chlorophyll in plants absorbs light to drive photosynthesis, converting light energy into chemical energy (Figure 1B) [39]. Inspired by this nontoxic, biocompatible magnesium‐based photochemistry, we aim to utilize nontoxic light main‐group metal photochemistry for the synthesis of potential drugs.

Our lab has been focusing on the development of biocompatible, inexpensive metal‐based photochemical systems by assembling readily available metal salts and inexpensive coordinating chemicals in situ [40, 41, 42, 43]. This strategy does not involve the pre‐preparation of photocatalysts and can be rapidly tuned by changing the metal salts and coordinating chemicals. Thus, we hope to leverage this in situ assembly strategy to unlock practical light main‐group metal photochemistry for the synthesis of potential drugs. Given that current nitroaromatic‐based drugs are mainly used to treat infectious diseases [44, 45, 46], while their anticancer counterparts remain under development, unlocking the potential of such compounds for anticancer therapy using light main‐group metal photochemistry is highly desirable (Figure 1C).

In light of these hypotheses, we rapidly developed an unprecedented lithium‐based visible‐light photochemical platform by assembling inexpensive LiNO_3_, methoxyacetic acid (a weak Brønsted acid), and aniline as the feedstock. This system enables the generation of nitro radicals for the efficient and selective C(sp^2^)–H nitration of various anilines, yielding nitroaromatic‐based products with promising antitumor activity. Notably, replacement of Li with Mg allows for the direct use of lower‐energy green light, further highlighting the biocompatibility of this protocol (Figure 1D).

## 2 Results and Discussion

**FIGURE 2:**
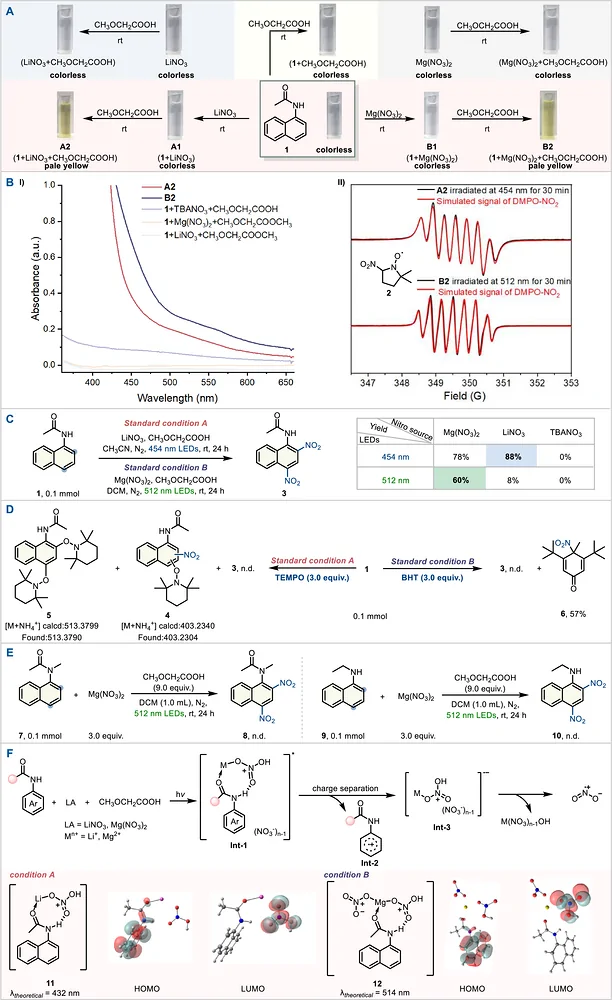
Establishment of the Li or Mg‐based photochemical nitration systems. (A) Ground‐state interactions among LiNO_3_ or Mg(NO_3_)_2_, **1**, and methoxyacetic acid. (B) UV–vis and EPR experiments. (C) Selective dinitration of aniline C(sp^2^)−H bonds using Li or Mg‐based photochemical systems. (D) Radical trapping experiments. (E) Necessity of the amide group with an intact N−H bond for the dinitration. (F) DFT studies on the Li or Mg‐mediated nitro radical generation.

We began our study by probing the ground‐state interactions among the main‐group metal (Li or Mg) nitrate salts, an amide arene substrate **1**, and the weak acid methoxyacetic acid via visual inspection (Figure 2A). Individual solutions of Mg(NO_3_)_2_, LiNO_3_, methoxyacetic acid, or their binary mixtures were colorless at room temperature. In contrast, mixing the amide substrate **1** with either Mg(NO_3_)_2_ or LiNO_3_ in the presence of methoxyacetic acid resulted in an immediate color change from colorless to pale yellow. The resulting Li‐based mixture (denoted as **A2**) and Mg‐based mixture (**B2**) both exhibited distinct absorption in the visible region (Figure 2B, I and Figure S9). Subsequently, **A2** and **B2** were irradiated with blue light (454 nm) and green light (512 nm) for 30 min, respectively. In both systems, the generation of NO_2_ radicals was confirmed by EPR spectroscopy (Figure 2B, II). Then, we extended the irradiation time to 24 h, and the selective dinitration of **1** could be achieved in satisfactory yields under both the Li‐based and Mg‐based photochemical systems [47, 48, 49, 50, 51, 52, 53, 54, 55, 56, 57, 58, 59, 60]. Using TBANO_3_ to replace LiNO_3_ or Mg(NO_3_)_2_ led to no nitration reactivity being observed, implying that the light main‐group metals Li or Mg are highly crucial for the generation of NO_2_ radicals (Figure 2C). The effect of reagent loading was further investigated by decreasing the amounts of methoxyacetic acid and LiNO_3_ or Mg(NO_3_)_2_. A gradual decrease in the yield of the desired product was observed under both the photochemical conditions (Tables S4 and S9). We proposed that the light main‐group metal‐based photochemical systems may be assembled through weak interactions among the amide arene substrate, LiNO_3_ or Mg(NO_3_)_2_, and methoxyacetic acid, and that high loading of methoxyacetic acid and LiNO_3_ or Mg(NO_3_)_2_ may favor the formation of the photoactive species to obtain the corresponding target products with high yield. Furthermore, the addition of the radical scavenger 2,6‐di‐tert‐butyl‐4‐methylphenol (BHT) or 2,2,6,6‐tetramethylpiperidin‐1‐oxyl (TEMPO) to the standard reaction completely suppressed the formation of the dinitrated product (Figure 2D). Notably, the BHT‐NO_2_ adduct **6** was successfully isolated in 59% yield from the inhibited reaction mixture, and the possible TEMPO‐**1** adduct **5** and TEMPO‐NO_2_ adduct **4** were detected by HRMS, providing additional conclusive evidence for the involvement of NO_2_ radicals and arene radical cations in the light main‐group metal photochemical systems.

We subsequently investigated the role of the amide group in the radical dinitration transformation (Figure 2E). When the amide N−H bond was *N*‐methylated (substrate **7**), no dinitration product was observed under the Mg‐based photochemical system. Similarly, removal of the amide carbonyl group (substrate **9**) also resulted in no formation of the dinitrated product. These results unambiguously demonstrated that the amide group with an intact N−H bond is essential for the formation of the light main‐group metal‐based photoactive species, serving as a crucial group to coordinate the main‐group metal center and enable subsequent visible light absorption and radical generation. Then, the possible light main‐group metal‐based photoactive species generated in situ was proposed (Figure 2F). Its core structure is constructed via the coordination of the amide group to Li/Mg and the hydrogen bonding between the N−H bond and protonated NO_3_^−^, which enables light absorption and ligand‐to‐ligand charge transfer (LLCT) to yield arene radical cations and NO_2_ radicals. DFT calculations evidenced that the ground‐state main‐group metal‐based species **11** and **12** exhibit the ability to absorb blue light and green light, respectively, which are in excellent agreement with our experimental excitation wavelengths and UV–vis absorption data. Frontier molecular orbital analysis showed that the highest occupied molecular orbital (HOMO) is predominantly localized on the arene substrate, while the lowest unoccupied molecular orbital (LUMO) is centered on protonated NO_3_^−^, confirming the validity of the LLCT to generate radical species mediated by Li or Mg.

**FIGURE 3:**
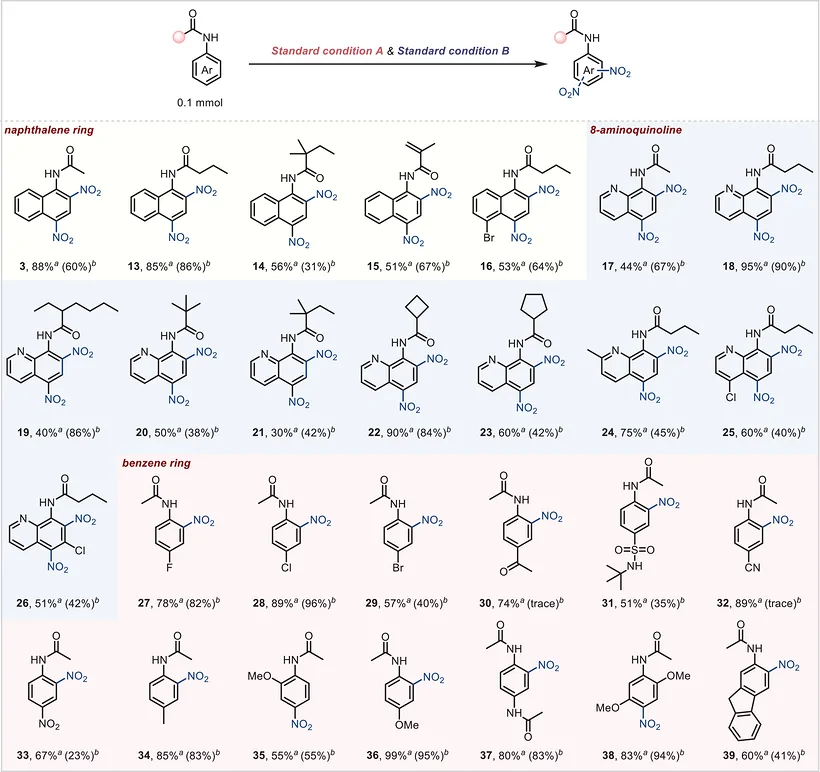
Substrate scope of anilines. *^a^* Standard condition A: substrate (0.1 mmol), CH_3_OCH_2_COOH (9.0 equiv.), LiNO_3_ (6.0 equiv.), CH_3_CN (1.0 mL), 454 nm LEDs, 24 h, N_2_, rt. *^b^* Standard condition B: substrate (0.1 mmol), CH_3_OCH_2_COOH (9.0 equiv.), Mg(NO_3_)_2_ (3.0 equiv.), DCM (1.0 mL), 512 nm LEDs, 24 h, N_2_, rt. Unless otherwise identified, yields were isolated following purification via silica column chromatography.

Having established the Li‐based and Mg‐based photochemical nitration of amide‐substituted arenes, we then systematically evaluated the generality and functional group compatibility of the two photochemical systems (Figure 3). All tested amide arenes delivered good to excellent yields under at least one of the two light main‐group metal‐based photochemical systems, which exhibited complementary reactivity, thus being compatible with naphthalene (**3**–**16**), 8‐aminoquinoline (**17**–**26**), and benzene‐derived aromatic amide substrates (**27**–**39**), and accommodating a wide range of electron‐donating (alkyl (**34**), methoxy (**35**, **36**, **38**), bis‐amide (**37**)) and electron‐withdrawing groups (halides (**27**–**29**), ketones (**30**), sulfonamide (**31**), nitrile (**32**), and pre‐existing nitro groups (**33**)). Importantly, nitration products **28**, **36**, and **38** were obtained in excellent to near‐quantitative yields under both protocols (**28**: 89% (Li) vs 96% (Mg); **36**: 99% vs 95%; **38**: 83% vs 94%). The two photochemical systems showed different sensitivities toward electron‐withdrawing groups. Aniline derivatives bearing ketone (**30**) and nitrile (**32**) groups exhibited significantly decreased reactivity under the Mg‐based photochemical system, affording only trace amounts of the desired products, whereas the Li‐based photochemical system enabled these transformations to proceed efficiently with good yields (74% and 88%, respectively). These results indicated that the electron‐withdrawing effects of electron‐deficient substituents exerted a stronger influence on the Mg‐based photochemical system than on the Li‐based system. This difference may be attributed to the lower‐energy green light employed in the Mg‐based photochemical system, which may provide insufficient driving force for the activation of more electron‐deficient substrates. Regarding the influence of steric effects, we found that compared with naphthylamine derivative **3** bearing an acetyl group, naphthylamine derivative **14** containing the sterically bulkier 2‐methylbutanoyl group delivered decreased yields under both photochemical conditions. Additionally, the influence of substitution patterns on the aromatic ring of aniline was also explored. For monosubstituted anilines, ortho‐ (**35**) and para‐substituted (**27**–**34**, **36**–**37**) substrates selectively afforded mononitrated products; for multisubstituted anilines (**38** and **39**), the mononitrated products could also be selectively obtained in moderate to good yields.

**FIGURE 4:**
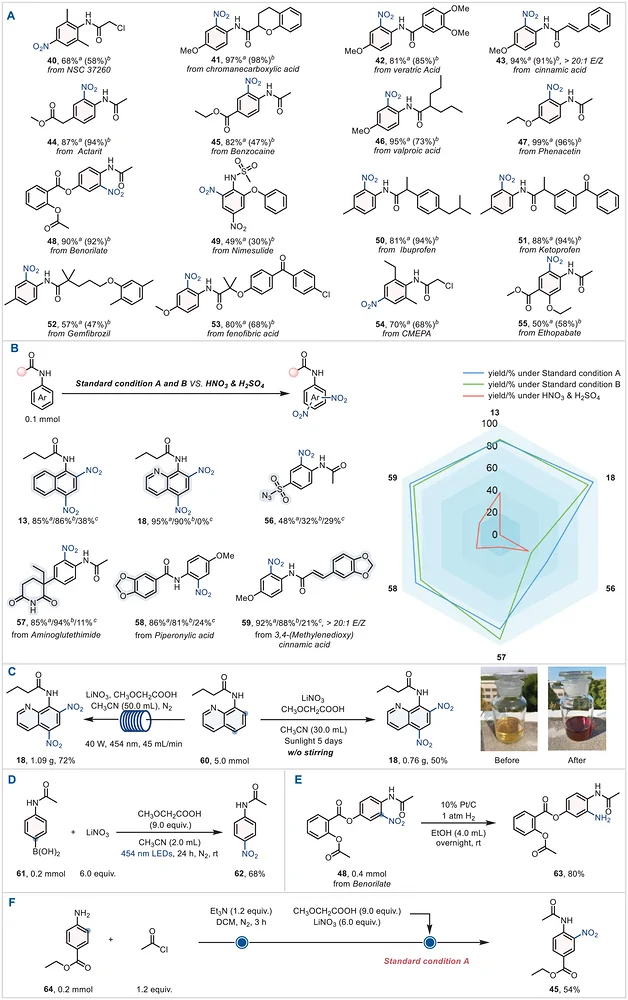
Synthetic expansion. (A) Late‐stage functionalization. (B) Our methods versus traditional strong acid conditions. (C) Gram‐scale synthesis using continuous flow and solar irradiation. (D) Nitration of arylboronic acid under the Li‐based photochemical system. (E) Reduction of the nitro group. (F) One‐pot synthesis. *^a^* Standard condition A: substrate (0.1 mmol), CH_3_OCH_2_COOH (9.0 equiv.), LiNO_3_ (6.0 equiv.), CH_3_CN (1.0 mL), 454 nm LEDs, 24 h, N_2_, rt. *^b^* Standard condition B: substrate (0.1 mmol), CH_3_OCH_2_COOH (9.0 equiv.), Mg(NO_3_)_2_ (3.0 equiv.), DCM (1.0 mL), 512 nm LEDs, 24 h, N_2_, rt. *^c^* Condition C: substrate (0.1 mmol), HNO_3_ (6.0 equiv.), H_2_SO_4_ (6.0 equiv.), DCM (1.0 mL), 3 h, N_2_, rt. Unless otherwise identified, yields were isolated following purification via silica column chromatography. n.d., not detected.

To demonstrate the synthetic utility of the two light main‐group metal photochemical platforms, a diverse array of complex bioactive molecules and natural product derivatives were subjected to the nitration protocol (Figure 4A). The mild reactions without the use of strong acids enabled good functional group tolerance, highlighting the potential of these methods for late‐stage functionalization of complex molecules. Amide arenes derived from commercial drugs (Actarit (**44**), Benzocaine (**45**), valproic acid (**46**), Phenacetin (**47**), Benorilate (**48**), Nimesulide (**49**), Ibuprofen (**50**), Ketoprofen (**51**), Gemfibrozil (**52**), fenofibric acid (**53**), Ethopabate (**55**)) as well as from naturally occurring scaffolds (NSC37260 (**40**), chromanecarboxylic acid (**41**), veratric acid (**42**), cinnamic acid (**43**), CMEPA (**54**)) were all well‐tolerated, affording the corresponding nitrated products in generally good to excellent yields.

Notably, the two complementary nitration conditions allowed fine‐tuning of the reactivity: for example, while **41**, **43**, **47**, **48**, and **51** gave high yields under both protocols (≥ 88% in each case), the drug derivatives such as **45** and **49** exhibited a clear preference, with significantly improved yields upon switching from the Mg‐based to the Li‐based conditions (**45**: 47% → 82%; **49**: 30% → 49%). Conversely, the Ibuprofen analogue **50** showed a slightly higher yield under the Mg‐based conditions (94% vs 81% under Li‐based). These results underscore the robustness and functional‐group tolerance of the two light main‐group metal photochemical platforms, enabling the direct, photocatalytic late‐stage diversification of structurally complex pharmaceuticals and natural products without the need for tedious prefunctionalization.

We next performed a direct comparative study between our mild visible‐light photochemical nitration conditions using light main‐group metals and classical concentrated HNO_3_/H_2_SO_4_ thermal nitration [61], employing representative amide arenes bearing diverse high‐value skeletons and functional groups (Figure 4B). The radar plot results clearly demonstrate the superiority of our protocol. For all tested amide arenes—including *N*‐heterocycles (**18**), fused aromatics (**13**), strong acid‐sensitive methylenedioxy groups (**58**), redox‐active sulfonyl azide (**56**), and drug‐derived complex scaffolds (**57**–**59**)—both of our light main‐group metal photochemical platforms furnished the target (di)nitrated products in good to excellent yields. In sharp contrast, the traditional strong acid nitration system afforded only trace amounts of product for all examined substrates. This direct comparison further validates the exceptional chemoselectivity, functional group tolerance, and mildness of our main‐group metal photochemical systems, offering a sustainable alternative to conventional nitration methods.

To validate the practical utility, broad applicability, and downstream synthetic value of the unprecedented Li‐based photocatalytic nitration system, we conducted several synthetic application studies. The scalability was demonstrated by gram‐scale continuous‐flow synthesis, which delivered 1.09 g of product **18** in 72% yield on a 5.0 mmol scale under 454 nm LEDs irradiation (Figure 4C, left). The reaction also proceeds efficiently under direct natural sunlight without external stirring, affording the desired product in 50% yield, highlighting its robustness, operational simplicity, and sustainability for synthesis (Figure 4C, right). Furthermore, the Li‐based system could be extended to the selective nitration of aryl boronic acid **61** in 68% yield, further expanding the substrate scope (Figure 4D). In addition, the nitrated products could be readily diversified into high‐value synthetic building blocks [62, 63, 64, 65, 66, 67, 68, 69, 70, 71, 72, 73, 74, 75]. For instance, the nitro group in the benorilate‐derived product **48** was cleanly reduced to aniline **63** in 80% yield under mild hydrogenation conditions, demonstrating excellent downstream synthetic utility (Figure 4E). To further improve synthetic efficiency, we developed a streamlined one‐pot, two‐step protocol starting from simple anilines: the amide coupling intermediate was directly nitrated under the Li‐based system without isolation or purification, furnishing product **45** in 54% overall yield, thus enhancing step economy and underscoring the practicality of this method for the rapid synthesis of functionalized nitroarenes (Figure 4F).

**FIGURE 5:**
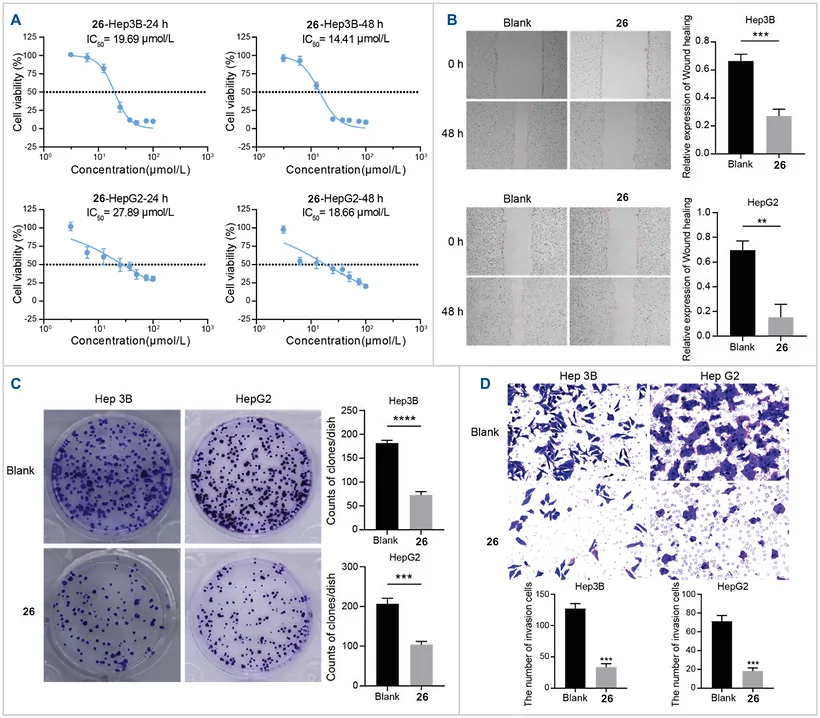
Antineoplastic assessment of 26 derived from the Li or Mg‐based photochemical platform. (A) Viability of Hep3B and HepG2 cells following 24 and 48 h treatment with **26**, as measured by CCK‐8 assay. Dose‐response curves are shown, with IC_50_ values indicated. (B) Effects of **26** on HCC cell migration assessed by wound healing assay. Representative images of wound areas at 0 and 48 h are displayed (left), along with quantification of wound closure rates (right). (C) Colony formation assay representative images (left) and quantitative analysis of the number of colonies formed (right). (D) Transwell invasion assay demonstrating the inhibitory effect of **26** on cell invasion. Representative micrographs of invaded cells stained with crystal violet are presented (top), together with counts of invaded cells per field (bottom). Data are expressed as mean ± SD from three independent experiments. ^**^ *p* < 0.01, ^***^ *p* < 0.001, ^****^ *p* < 0.0001 vs. control group.

With the two biocompatible light main‐group metal photochemical nitration protocols in hand, we finally examined the desirable anti‐tumor activity of our nitration products. We found that dinitration product **26** possesses excellent antitumor activity. Initial viability assessment via the CCK‐8 assay showed that **26** suppressed the growth of two human HCC cell lines, Hep3B and HepG2, in a manner dependent on both treatment duration and concentration. This growth‐inhibitory activity was reflected in the calculated IC_50_ values, which declined from 19.69 µmol/L for Hep3B and 27.89 µmol/L for HepG2 at 24 h to 14.41 µmol/L and 18.66 µmol/L, respectively, after 48 h of exposure (Figure 5A), demonstrating increased potency with longer treatment.

To gain deeper insight into **26**’s anti‐cancer potential, we investigated its impact on key malignant behaviors: migration, clonogenic survival, and invasion. A wound healing assay revealed substantially impeded cell motility, as wounds in **26**‐treated cultures closed much more slowly than those in control wells after 48 h (Figure 5B). Furthermore, **26** severely compromised long‐term proliferative capacity, evidenced by a pronounced reduction in both the number and size of cell colonies in a clonogenic assay (Figure 5C). Crucially, **26** also potently inhibited the invasive potential of HCC cells, a critical determinant of metastasis. In a Transwell‐based invasion assay, significantly fewer cells penetrated the Matrigel‐coated membrane following **26** treatment (Figure 5D). Together, results from these complementary functional analyses strongly indicate that **26** exerts substantial anti‐HCC effects by simultaneously targeting multiple oncogenic processes—proliferation, migration, clonogenicity, and invasion—underscoring its promise for further therapeutic exploration.

To further evaluate the broad‐spectrum antitumor activity of the dinitration product, we subsequently tested the inhibitory activities of **16** and **18** against KYSE30 (esophageal carcinoma cells). In the presence of **18**, the survival rate of KYSE30 cells was about 1%, whereas HET‐1A normal human cells accessibly exhibited a survival rate of 52%. Although the survival rate of HET‐1A normal human cells was slightly diminished in the presence of **16**, the survival rate of KYSE30 cells still reached about 1%. The significant mortality observed in cancer cells, combined with the favorable survival rate of normal cells, suggests that **16** and **18** may provide a way for novel therapeutic strategies in the treatment of esophageal cancer (Figure S31).

## 3 Conclusions

In conclusion, we have developed an in situ‐assembled strategy to unlock an unprecedented light Li‐based photochemical nitration system. This catalytic assembly is generated from biocompatible LiNO_3_, methoxyacetic acid, and aniline substrates, and absorbs visible light to efficiently generate nitro radicals. The modular nature of this system allows straightforward expansion to the Mg‐based platform by replacing LiNO_3_ with Mg(NO_3_)_2_, affording a milder Mg‐based photochemical system that functions under green light to produce nitro radicals. Both photochemical platforms enable the selective mono‐ and di‐nitration of aniline C(sp^2^)–H bonds. Notably, the dinitrated products show promise as antitumor candidates. Further investigations into the development and application of biocompatible light main‐group metal‐based photochemical systems are ongoing in our laboratory.

## Author Contributions

**Qian Wan**: methodology, data curation, investigation, writing – original draft, writing – review and editing, conceptualization. **Jiali Zhu**: writing – original draft, writing – review and editing, methodology, data curation, investigation, conceptualization. **Shanshuo Liu**: writing – original draft, writing – review and editing, investigation, methodology, data curation, conceptualization. **Luchuan Ou**: data curation, validation, methodology. **Huizhen Wang**: methodology, data curation, validation. **Yu Lan**: supervision, funding acquisition, resources, conceptualization. **Zhou Dong**: methodology, data curation, validation. **Yihang Bai**: methodology, data curation, validation. **Wei Yang**: methodology, data curation, validation. **Shihan Liu**: methodology, data curation, validation, funding acquisition. **Yan Qiao**: investigation, supervision, resources, conceptualization. **Shi‐Jun Li**: investigation, supervision, resources, writing – original draft, writing – review and editing, conceptualization. **Zhigang Ren**: investigation, supervision, resources, writing – original draft, writing – review and editing, conceptualization. **Linbin Niu**: investigation, supervision, funding acquisition, resources, writing – original draft, writing – review and editing, conceptualization.

## Conflicts of Interest

The authors declare no conflicts of interest.

## Supporting information




**Supporting file**: advs77873‐sup‐0001‐SuppMat.docx.

## Data Availability

The data that supports the findings of this study are available in the supplementary material of this article.
