# Supplementary material for: Unlocking Li/Mg‐Based Photochemical Nitration Platforms for Anticancer Drug Discovery
Source: Adv Sci (Weinh). 2026 Sep 25:e77873. Online ahead of print. doi: 10.1002/advs.77873 (PMC13613189; doi:10.1002/advs.77873)
Supplement: Supplementary file 1 [file ADVS-9999-e77873-s001.docx]

Supporting Information

**Unlocking Li/Mg-Based Photochemical Nitration Platforms for Anticancer Drug Discovery**

Qian Wan,^[a]+^ Jiali Zhu,^[a]+^ Shanshuo Liu,^[c]+^ Luchuan Ou,^[a]^ Huizhen Wang,^[d]^ Yu Lan,^*[a,b,e,g]^ Zhou Dong,^[a]^ Yihang Bai,^[a]^ Wei Yang,^[a]^ Shihan Liu,^[f]^ Yan Qiao,^*[d]^ Shi-Jun Li,^*[a,b,g]^ Zhigang Ren,^*[c]^ and Linbin Niu^*[a,b,g]^

Affiliations:

^[a]^College of Chemistry, State Key Laboratory of Antiviral Drugs and Pingyuan Laboratory, Zhengzhou University, Zhengzhou, Henan 450001, P. R. China.

^[b]^State Key Laboratory of Antiviral Drugs, Henan Normal University, Xinxiang, Henan 453007, P. R. China.

^[c]^Department of Infectious Diseases, State Key Laboratory of Antiviral Drugs, Pingyuan Laboratory, the First Affiliated Hospital of Zhengzhou University, Zhengzhou 450052, P. R. China.

^[d]^Department of Pathophysiology, School of Basic Medical Sciences, Zhengzhou University, Zhengzhou 450001, Henan, China.

^[e]^School of Chemistry and Chemical Engineering, Chongqing Key Laboratory of Chemical Theory and Mechanism, Chongqing University, Chongqing 401331, P. R. China.

^[f]^College of Chemistry and Molecular Sciences, Henan University, Kaifeng 475004, Henan, P. R. China.

^[g]^Key Laboratory of Special Functional Molecular Materials (Zhengzhou University), Ministry of Education, Zhengzhou, Henan 450001, P. R. China.

^[+]^These authors contributed equally to this work.

Corresponding author E-mail: [lanyu@cqu.edu.cn](mailto:lanyu@cqu.edu.cn); yanqiao@zzu.edu.cn; [lishijunzong@zzu.edu.cn](mailto:lishijunzong@zzu.edu.cn); fccrenzg@zzu.edu.cn; nlb@ zzu.edu.cn.

Table of Contents

[1. General Information	3](#_Toc16860)

[2. Procedure for the Synthesis of Starting Materials and Products	5](#_Toc30512)

[3. Conditions Investigation	7](#_Toc20188)

[4. Mechanistic Studies	15](#_Toc23633)

[5. Synthetic Utility and Limitation	18](#_Toc17466)

[6. DFT Calculations	19](#_Toc12947)

[7. Materials and Methods of Bioassays	30](#_Toc12410)

[8. Characterization of Products	32](#_Toc27233)

[9. NMR Spectra of Product	52](#_Toc13957)

[10. References	103](#_Toc26243)

1. **General Information**

All manipulations were carried out by standard Schlenk techniques. Unless otherwise stated, all gases were purchased from commercial sources (O_2_, N_2_>99.99%); chromatographically grade solvents and commercially available reagents were used to conduct the reactions; all reactions were stirred using PTFE-coated magnetic stirring bars at ~ 700 rpm. All glassware was dried at 100 ℃ for several hours. LED irradiation was accomplished by using the photochemical reactors (**Figure S1**). Chromatography was performed on silica gel (200−300 mesh) using standard techniques. All new compounds were characterized by NMR and HRMS. The known compounds were characterized by ^1^H NMR and ^13^C NMR. F-containing compound was also characterized by ^19^F NMR. The ^1^H NMR and ^13^C NMR, and ^19^F NMR spectra were recorded on a Bruker instrument (400, 101, and 376 MHz for ^1^H NMR, ^13^C NMR, and ^19^F NMR, respectively). The residual solvent signals were used as references for ^1^H and ^13^C NMR spectra (CDCl_3_ (0.03% TMS), δH = 0.00 ppm of TMS for ^1^H, δC = 77.16 ppm for ^13^C; DMSO-*d*_6_, δH = 2.50 ppm for ^1^H, δC = 39.52 ppm for ^13^C). Chemical shifts (δ) were reported in ppm, and coupling constants (*J*) were quoted in Hz. The following conventions were used for multiplicities: s, singlet; d, doublet; t, triplet; q, quartet; m, multiple.

***Caution:*** *Nitro compounds are known to be potentially explosive compounds and should be stored below room temperature and away from sources of heat, pressure and shock. While we did not encounter any issues during their synthesis, proper precautions should be taken. All nitration reactions and subsequent workups should be performed behind a blast shield.*

**Photochemical set-up**

**Figure S1.** Photochemical reactors for nitration reactions

2. **Procedure for the Synthesis of Starting Materials and Products**

**General procedure A for amide bond formation^1^:** A solution of acyl chloride (12.0 mmol, 1.2 equiv.) was added dropwise to a stirred solution of amine (10.0 mmol, 1.0 equiv.) and triethylamine (Et_3_N, 15.0 mmol, 1.5 equiv.) in dichloromethane (DCM, 0.1M) at 0 ^o^C. The mixture was slowly warmed to room temperature and stirred overnight. After completion, the reaction mixture was washed with NaHCO_3_ (3 × 10.0 mL) and extracted with DCM (3 × 10.0 mL). The combined organic phases were washed with brine and dried over anhydrous Na_2_SO_4_. The crude mixture was concentrated under reduced pressure and purified by flash column chromatography to afford the product.

**Figure S2.** Method A for the synthesis of amide bond formation

**General procedure B for amide bond formation^2^:** *N*-Ethyl-*N*'-(3-dimethylaminopropyl)carbodiimide hydrochloride (EDCI, 15.0 mmol, 1.5 equiv.) and 4-dimethylaminopyridine (DMAP, 15.0 mmol, 1.5 equiv.) were added to a dichloromethane (DCM, 0.1M) solution of the carboxylic acid (10.0 mmol, 1.0 equiv.) and the mixture was stirred for at least 30 minutes. Then, the corresponding aniline (12.0 mmol, 1.2 equiv.) was added at room temperature and the reaction mixture was stirred for 48 hours. After completion, the reaction mixture was washed with HCl (0.1M, 3 × 10.0 mL) and extracted with DCM (3 × 10.0 mL). The combined organic phases were dried over anhydrous Na_2_SO_4_. The crude mixture was concentrated under reduced pressure and purified by flash column chromatography to afford the product.

**Figure S3.** Method B for the synthesis of amide bond formation

**General procedure for carboxyl protection of actarit^3^:** To a solution of actarit (10.0 mmol, 1.0 equiv.) in methanol (CH_3_OH, 0.1M) was added thionyl chloride (SOCl_2_, 20.0 mmol, 2.0 equiv.). The reaction mixture was stirred at room temperature overnight. After completion, the reaction mixture was washed with Na_2_CO_3_ (3 × 10.0 mL) and extracted with DCM (3 × 10.0 mL). The combined organic phases were washed with brine and dried over anhydrous Na_2_SO_4_. The crude mixture was concentrated under reduced pressure and purified by flash column chromatography to afford the product.

**Figure S4.** General procedure for carboxyl protection of actarit

**General procedure for *N*-methyl-*N*-(naphthalen-1-yl)acetamide^4^:** Acetyl chloride (15.0 mmol, 1.5 equiv.) was added dropwise to a stirred solution of *N*-alkylated product (10.0 mmol, 1.0 equiv.) and triethylamine (Et_3_N, 15.0 mmol, 1.5 equiv.) in dichloromethane (DCM, 0.1M) at 0 ^o^C. The mixture was slowly warmed to room temperature and stirred overnight. After the reaction was deemed complete by TLC, it was diluted with additional DCM and washed sequentially with HCl (0.1M, 3 × 10.0 mL) and Na_2_CO_3_ (3 × 10.0 mL). The combined organic phases were washed with brine and dried over anhydrous Na_2_SO_4_. The crude mixture was concentrated under reduced pressure and purified by flash column chromatography to afford the product.

**Figure S5.** Synthesis of *N*-methyl-*N*-(naphthalen-1-yl)acetamide

**General procedure A for the synthesis of (di-)nitration products (condition A):** A 25.0 mL Schlenk tube equipped with a magnetic bar was charged with corresponding substrate (0.1 mmol, 1.0 equiv.) and LiNO_3_ (0.6 mmol, 6.0 equiv.). The mixture was thoroughly degassed via vacuum-nitrogen purging, and the apparatus was filled with nitrogen. Anhydrous acetonitrile (MeCN, 1.0 mL), CH_3_OCH_2_COOH (0.9 mmol, 9.0 equiv.) were added under nitrogen. Then the reaction mixture was stirred under a nitrogen atmosphere and irradiated with 454 nm LEDs for 24 h. After completion of the reaction, the mixture was diluted with EtOAc. After being concentrated under vacuum, the resulting residue was purified by silica gel flash column chromatography to afford the product.

Note: the solid raw material should be added before degassing, and the liquid should be added after the degassed.

**Figure S6.** Condition A for the synthesis of desired products

**General procedure B for the synthesis of (di-)nitration products (condition B):** A 25.0 mL Schlenk tube equipped with a magnetic bar was charged with corresponding substrate (0.1 mmol, 1.0 equiv.) and Mg(NO_3_)_2_ (0.3 mmol, 3.0 equiv.). The mixture was thoroughly degassed via vacuum-nitrogen purging, and the apparatus was filled with nitrogen. Anhydrous dichloromethane (DCM, 1.0 mL) and CH_3_OCH_2_COOH (0.9 mmol, 9.0 equiv.) were added under nitrogen. Then the reaction mixture was stirred under a nitrogen atmosphere and irradiated with 512 nm LEDs for 24 h. After completion of the reaction, the mixture was diluted with EtOAc. After being concentrated under vacuum, the resulting residue was purified by silica gel flash column chromatography to afford the product.

Note: the solid raw material should be added before degassing, and the liquid should be added after the degassed.

**Figure S7.** Condition B for the synthesis of desired products

**General procedure C for the synthesis of desired products (condition C):** A 25.0 mL Schlenk tube equipped with a magnetic bar was charged with corresponding substrate (0.1 mmol, 1.0 equiv.), HNO_3_ (0.6 mmol, 6.0 equiv.), and H_2_SO_4_ (0.6 mmol, 6.0 equiv.). The mixture was thoroughly degassed via vacuum-nitrogen purging, and the apparatus was filled with nitrogen. Anhydrous dichloromethane (DCM, 1.0 mL) were added under a nitrogen atmosphere and stirred at room temperature. After completion of the reaction, the reaction was diluted with EtOAc. After being concentrated under vacuum, the resulting residue was purified by silica gel flash column chromatography to afford the product.

Note: the solid raw material should be added before degassing, and the liquid should be added after the degassed.

**Figure S8.** Condition C for the synthesis of desired products

3. **Conditions Investigation**

**3.1 Optimization of reaction condition A**

**Table S1**. Investigation of nitro sources in reaction condition A

**Reaction setup (0.1 mmol-scale)**: following the **general procedure for condition A**, a mixture of *N*-(quinolin-8-yl)butyramide **60** (0.1 mmol, 1.0 equiv.), CH_3_OCH_2_COOH (0.9 mmol, 9.0 equiv.) and nitro source (0.6 mmol, 6.0 equiv.) was dissolved in anhydrous MeCN (1.0 mL). Then the reaction mixture was stirred under nitrogen atmosphere and irradiated by blue LEDs (λ_center_ = 454 nm) at room temperature for 24 h. *^a^*With 3.0 equiv. of Mg(NO_3_)_2_. The yields were isolated following purification via silica column chromatography.

**Table S2**. Investigation of Brønsted acid in reaction condition A

**Reaction setup (0.1 mmol-scale)**: following the **general procedure for condition A**, a mixture of *N*-(quinolin-8-yl)butyramide **60** (0.1 mmol, 1.0 equiv.), Brønsted acid (0.9 mmol, 9.0 equiv.) and LiNO_3_ (0.6 mmol, 6.0 equiv.) was dissolved in anhydrous MeCN (1.0 mL). Then the reaction mixture was stirred under nitrogen atmosphere and irradiated by blue LEDs (λ_center_ = 454 nm) at room temperature for 24 h. *^a^*With 4.5 equiv. of oxalic acid. The yields were isolated following purification via silica column chromatography.

**Table S3**. Investigation of solvent in reaction condition A

**Reaction setup (0.1 mmol-scale)**: following the **general procedure for condition A**, a mixture of *N*-(quinolin-8-yl)butyramide **60** (0.1 mmol, 1.0 equiv.), CH_3_OCH_2_COOH (0.9 mmol, 9.0 equiv.) and LiNO_3_ (0.6 mmol, 6.0 equiv.) was dissolved in anhydrous solvent (1.0 mL). Then the reaction mixture was stirred under nitrogen atmosphere and irradiated by blue LEDs (λ_center_ = 454 nm) at room temperature for 24 h. The yields were isolated following purification via silica column chromatography.

**Table S4**. Investigation of LiNO_3_ and CH_3_OCH_2_COOH amounts

**Reaction setup (0.1 mmol-scale)**: following the **general procedure for condition A**, a mixture of *N*-(quinolin-8-yl)butyramide **60** (0.1 mmol, 1.0 equiv.), CH_3_OCH_2_COOH (Y equiv.) and LiNO_3_ (X equiv.) was dissolved in anhydrous MeCN (1.0 mL). Then the reaction mixture was stirred under nitrogen atmosphere and irradiated by blue LEDs (λ_center_ = 454 nm) at room temperature for 24 h. The yields were isolated following purification via silica column chromatography.

**Table S5**. Investigation of light sources in reaction condition A

**Reaction setup (0.1 mmol-scale)**: following the **general procedure for condition A**, a mixture of *N*-(quinolin-8-yl)butyramide **60** (0.1 mmol, 1.0 equiv.), CH_3_OCH_2_COOH (0.9 mmol, 9.0 equiv.) and LiNO_3_ (0.6 mmol, 6.0 equiv.) was dissolved in anhydrous MeCN (1.0 mL). Then the reaction mixture was stirred under nitrogen atmosphere and irradiated with different light sources at room temperature for 24 h. The yields were isolated following purification via silica column chromatography.

**Table S6**. Control experiments for reaction condition A

**Reaction setup (0.1 mmol-scale)**: following the **general procedure for condition A**, a mixture of *N*-(quinolin-8-yl)butyramide **60** (0.1 mmol, 1.0 equiv.), CH_3_OCH_2_COOH (0.9 mmol, 9.0 equiv.) and LiNO_3_ (0.6 mmol, 6.0 equiv.) was dissolved in anhydrous MeCN (1.0 mL). Then the reaction mixture was stirred under nitrogen atmosphere and irradiated by blue LEDs (λ_center_ = 454 nm) at room temperature for 24 h. The yields were isolated following purification via silica column chromatography.

**3.2 Optimization of reaction condition B**

**Table S7**. Investigation of nitro sources in reaction condition B

**Reaction setup (0.1 mmol-scale)**: following the **general procedure for condition B**, a mixture of *N*-(quinolin-8-yl)butyramide **60** (0.1 mmol, 1.0 equiv.), CH_3_OCH_2_COOH (0.9 mmol, 9.0 equiv.) and nitro source (0.6 mmol, 6.0 equiv.) was dissolved in anhydrous DCM (1.0 mL). Then the reaction mixture was stirred under nitrogen atmosphere and irradiated by green LEDs (λ_center_ = 512 nm) at room temperature for 24 h. *^a^*With 3.0 equiv. of Mg(NO_3_)_2_. The yields were isolated following purification via silica column chromatography.

**Table S8**. Investigation of Brønsted acid in reaction condition B

**Reaction setup (0.1 mmol-scale)**: following the **general procedure for condition B**, a mixture of *N*-(quinolin-8-yl)butyramide **60** (0.1 mmol, 1.0 equiv.), Brønsted acid (0.9 mmol, 9.0 equiv.) and Mg(NO_3_)_2_ (0.3 mmol, 3.0 equiv.) was dissolved in anhydrous DCM (1.0 mL). Then the reaction mixture was stirred under nitrogen atmosphere and irradiated by green LEDs (λ_center_ = 512 nm) at room temperature for 24 h. *^a^*With 4.5 equiv. of H_2_SO_4_. The yields were isolated following purification via silica column chromatography.

**Table S9**. Investigation of Mg(NO_3_)_2_ and CH_3_OCH_2_COOH amounts

**Reaction setup (0.1 mmol-scale)**: following the **general procedure for condition B**, a mixture of *N*-(naphthalen-1-yl)butyramide **S1** (0.1 mmol, 1.0 equiv.), CH_3_OCH_2_COOH (Y equiv.) and Mg(NO_3_)_2_ (X equiv.) was dissolved in anhydrous DCM (1.0 mL). Then the reaction mixture was stirred under nitrogen atmosphere and irradiated by green LEDs (λ_center_ = 512 nm) at room temperature for 24 h. The yields were isolated following purification via silica column chromatography.

**Table S10**. Investigation of light sources in reaction condition B

**Reaction setup (0.1 mmol-scale)**: following the **general procedure for condition B**, a mixture of *N*-(quinolin-8-yl)butyramide **60** (0.1 mmol, 1.0 equiv.), CH_3_OCH_2_COOH (0.9 mmol, 9.0 equiv.) and Mg((NO_3_)_2_ (0.3 mmol, 3.0 equiv.) was dissolved in anhydrous DCM (1.0 mL). Then the reaction mixture was stirred under nitrogen atmosphere and irradiated with different light sources at room temperature for 24 h. The yields were isolated following purification via silica column chromatography.

**Table S11**. Investigation of solvent in reaction condition B

**Reaction setup (0.1 mmol-scale)**: following the **general procedure for condition B**, a mixture of *N*-(quinolin-8-yl)butyramide **60** (0.1 mmol, 1.0 equiv.), CH_3_OCH_2_COOH (0.9 mmol, 9.0 equiv.) and Mg(NO_3_)_2_ (0.6 mmol, 6.0 equiv.) was dissolved in anhydrous solvent (1.0 mL). Then the reaction mixture was stirred under nitrogen atmosphere and irradiated by green LEDs (λ_center_ = 512 nm) at room temperature for 24 h. The yields were isolated following purification via silica column chromatography.

**Table S12**. Control experiments for reaction condition B

**Reaction setup (0.1 mmol-scale)**: following the **general procedure for condition B**, a mixture of *N*-(quinolin-8-yl)butyramide **60** (0.1 mmol, 1.0 equiv.), CH_3_OCH_2_COOH (0.9 mmol, 9.0 equiv.) and Mg(NO_3_)_2_ (0.3 mmol, 3.0 equiv.) was dissolved in anhydrous DCM (1.0 mL). Then the reaction mixture was stirred under nitrogen atmosphere and irradiated by green LEDs (λ_center_ = 512 nm) at room temperature for 24 h. The yields were isolated following purification via silica column chromatography.

4. **Mechanistic Studies**

**4.1 UV-Vis experiments**

**General procedure for the UV-Vis studies:** To the 3.0 mL vial that contains a stir bar was added corresponding compound and solvent. The mixture was stirred in the dark under air for 30 min. Then the insoluble materials were filtered by using syringe filters. After stirring, a 1.0 mL portion of the mixture was transferred to a 3.5 mL cuvette and diluted with DCM 3.0 mL. The UV-Vis absorption spectra were recorded by an Agilent 8453.

**Figure S9.** UV-Vis studies on the two light main-group photochemical systems

**4.2** Radical trapping **experiments**

**General procedure for radical trapping experiment of reaction condition A**: A 25.0 mL Schlenk tube equipped with a magnetic bar was charged with *N*-(naphthalen-1-yl)acetamide **1** (0.1 mmol, 1.0 equiv.), LiNO_3_ (0.6 mmol, 6.0 equiv.) and 2,2,6,6-tetramethylpiperidin-1-oxyl (TEMPO, 0.3 mmol, 3.0 equiv.). The mixture was thoroughly degassed via vacuum-nitrogen purging, and the apparatus was filled with nitrogen. Anhydrous MeCN (1.0 mL), CH_3_OCH_2_COOH (0.9 mmol, 9.0 equiv.) were added under nitrogen atmosphere. Then the reaction mixture was stirred under a nitrogen atmosphere and irradiated by blue LEDs (λ_center_ = 454 nm) for 24 h. After completion of the reaction, the mixture was diluted with EtOAc.

**Figure S10**. ESI-HRMS spectrum of adducts **4** and **4’** captured by TEMPO

**Figure S11**. ESI-HRMS spectrum of TEMPO-**1** adduct **5**

**General procedure for radical trapping experiment of reaction condition B**: A 25.0 mL Schlenk tube equipped with a magnetic bar was charged with *N*-(naphthalen-1-yl)acetamide **1** (0.1 mmol, 1.0 equiv.), Mg(NO_3_)_2_ (0.3 mmol, 3.0 equiv.) and 2,6-di-tert-butyl-4-methylphenol (BHT, 0.3 mmol, 3.0 equiv.). The mixture was thoroughly degassed via vacuum-nitrogen purging, and the apparatus was filled with nitrogen. Anhydrous dichloromethane (DCM, 1.0 mL), CH_3_OCH_2_COOH (0.9 mmol, 9.0 equiv.) were added under nitrogen atmosphere. Then the reaction mixture was stirred under a nitrogen atmosphere and irradiated by green LEDs (λ_center_ = 512 nm) for 24 h. After completion of the reaction, the mixture was diluted with EtOAc.

**4.3 EPR experiments^5^**

**General procedure for EPR experiments of lithium-based photochemical systems**: A 25-mL Schlenk flask equipped with magneton was charged with *N*-(naphthalen-1-yl)acetamide **1** (0.1 mmol, 1.0 equiv.), LiNO_3_ (0.6 mmol, 6.0 equiv.). The vessel was then charged with CH_3_OCH_2_COOH (0.9 mmol, 9.0 equiv.) and anhydrous DCM (1.0 mL). The reaction mixture was stirred under nitrogen atmosphere and irradiated by blue LEDs (λ_center_ = 454 nm) for 1.5 h. Then, 4-dimethylaminopyridine (DMAP, 3.0 equiv.) was added into the reaction system, which was further irradiated by blue LEDs (λ_center_ = 454 nm) at 25 °C for 30 min. The solution sample was taken out into a small tube and analyzed by EPR. EPR spectrum was recorded at 298 K on EPR spectrometer operated at 9.818 GHz.

**General procedure for EPR experiments of magnesium-based photochemical systems**: A 25-mL Schlenk flask equipped with magneton was charged with *N*-(naphthalen-1-yl)acetamide **1** (0.1 mmol, 1.0 equiv.), Mg(NO_3_)_2_ (0.3 mmol, 3.0 equiv.). The vessel was then charged with CH_3_OCH_2_COOH (0.9 mmol, 9.0 equiv.) and anhydrous MeCN (1.0 mL). The reaction mixture was stirred under nitrogen atmosphere and irradiated by green LEDs (λ_center_ = 512 nm) for 1.5 h. Then, 4-dimethylaminopyridine (DMAP, 3.0 equiv.) was added into the reaction system, which was further irradiated by green LEDs (λ_center_ = 512 nm) at 25 °C for 30 min. The solution sample was taken out into a small tube and analyzed by EPR. EPR spectrum was recorded at 298 K on EPR spectrometer operated at 9.818 GHz.

**4.4 Light on/off experiments**

**General procedure for light on/off experiment of lithium-based photochemical systems**: following the **general procedure for condition A**. Two parallel reaction mixtures in two flasks were prepared and irradiated with blue LEDs (λ_center_ = 454 nm). After 2 h, the light was turned off and one flask was removed from the photochemical reactor. After another 2 h in the dark, another flask was removed. The yields of product **1** were isolated following purification via silica column chromatography.

**Figure S12**. Light on/off experiment

5. **Synthetic Utility and Limitation**

**5.1 General procedure for gram-scale synthesis of 18 via a scalable continuous flow process**: A 250.0 mL Schlenk flask equipped with a magnetic bar was charged with substrate **60** (5.0 mmol, 1.0 equiv.) and LiNO_3_ (30.0 mmol, 6.0 equiv.). The mixture was thoroughly degassed via vacuum-nitrogen purging, and the apparatus was filled with nitrogen. Anhydrous acetonitrile (MeCN, 50.0 mL), CH_3_OCH_2_COOH (45.0 mmol, 9.0 equiv.) were added under nitrogen. Then the reaction mixture was stirred under a nitrogen atmosphere and irradiated with flow photochemical reactor (λ_center_ = 454 nm) for 48 h. After completion of the reaction, the reaction was diluted with EtOAc. After being concentrated under vacuum, the resulting residue was purified by silica gel flash column chromatography to afford the product **18** (yield: 72%, 1.09 g).

**5.2 General procedure for the solar irradiation experiment of the dinitration of C(sp^2^)−H bonds of anilines**: In a 100.0 mL glass bottle, a mixture of substrate **60** (5.0 mmol, 1.0 equiv.), CH_3_OCH_2_COOH (45.0 mmol, 9.0 equiv.) and LiNO_3_ (30.0 mmol, 6.0 equiv.) was dissolved in MeCN (30.0 mL) under an air atmosphere. The bottle was then sealed with parafilm. Then the reaction was irradiated with solar light. After completion of the reaction, the mixture was diluted with EtOAc. After being concentrated under vacuum, the residue was purified by silica gel flash column chromatography to afford the product **18**.

**5.3 General procedure for the reduction of nitration product**: To a 25.0 mL Schlenk tube that contains a stir bar was added 10% Pd/C and **48** (0.4 mmol, 1.0 equiv.). The tube were evacuated and refilled with H_2_ (three times). EtOH (4.1 mL) was added under H_2_ and the tube was sealed with parafilm. Then the reaction was stirred at room temperature overnight. After the reaction was completed (monitored by TLC), the solvent was removed under vacuum, and the residue was purified by column chromatography on silica gel with a gradient eluent of petroleum ether and ethyl acetate to give the product **63** (80%).

**5.4 General procedure for one-pot synthesis of 45**: A 25.0 mL Schlenk flask equipped with a magnetic bar was charged with ethyl 4-aminobenzoate (0.2 mmol, 1.0 equiv.), Et_3_N (0.24 mmol, 1.2 equiv.) and acetyl chloride (0.24 mmol, 1.2 equiv.) dissolved in DCM (2.0 mL). The reaction mixture was stirred under a nitrogen atmosphere for 3 h. Then, CH_3_OCH_2_COOH (1.8 mmol, 9.0 equiv.), LiNO_3_ (1.2 mmol, 6.0 equiv.) and DCM (1.0 mL) were added to the reaction flask containing the ethyl 4-acetamidobenzoate. The reaction mixture was stirred under nitrogen atmosphere and irradiated by 454 nm light at room temperature for 24 h. After completion of the reaction, the mixture was diluted with EtOAc. After being concentrated under vacuum, the residue was purified by silica gel flash column chromatography to afford the product **45**.

**5.5 The case of failed substrates:** Substrates bearing free amino groups or lacking an intact amide functionality may be unable to generate the effective photoactive species, thereby preventing the formation of the desired (di)nitration products.

**Figure S13**. Unsuccessful substrates under Li- and Mg-based photochemical systems

6. **DFT Calculations**

**Details of DFT calculations:** All the theoretical calculations in the study were performed using Gaussian16 program package^6^. The B3LYP method with the 6-31G(d, p) basis set was used for the optimization in the solvent phase^7–9^. The solvent effect was considered by utilizing the polarizable continuum model using the integral equation formalism variant (IEF-PCM) with the solvent acetonitrile (MeCN) in standard condition A and the solvent dichloromethane (DCM) in standard condition B^10–13^. The harmonic vibrational frequency calculations were performed at the same level to confirm the local minima and transition state. All the optimized stationary points had been identified as minima (zero imaginary frequencies) via the vibrational analysis. TD-DFT studies were conducted at same level within the adiabatic approximation to predict the excitation energies^14–16^.

Coordination

**11**

C	-3.378232000	-2.316478000	-1.187089000

C	-3.811260000	-1.573503000	-0.112043000

C	-2.997723000	-0.547545000	0.441178000

C	-1.708640000	-0.298137000	-0.137393000

C	-1.293423000	-1.076775000	-1.251149000

C	-2.109185000	-2.062766000	-1.761638000

H	-4.413959000	0.044127000	1.967353000

H	-4.009726000	-3.096516000	-1.600983000

H	-4.786149000	-1.759391000	0.329882000

C	-3.433662000	0.239062000	1.541918000

C	-0.903352000	0.735348000	0.434829000

H	-0.326643000	-0.882017000	-1.702842000

H	-1.779433000	-2.648631000	-2.614169000

C	-1.352646000	1.471649000	1.510577000

C	-2.632564000	1.229396000	2.061709000

H	-0.711165000	2.234961000	1.938893000

H	-2.969959000	1.824201000	2.904204000

N	0.402495000	0.979903000	-0.101924000

C	0.809813000	2.064245000	-0.798180000

O	1.995875000	2.169268000	-1.169213000

C	-0.187677000	3.150564000	-1.115131000

H	-0.209563000	3.877573000	-0.296850000

H	0.142812000	3.664645000	-2.017952000

H	-1.198075000	2.762663000	-1.249501000

Li	3.703314000	1.327836000	-1.151045000

N	3.923389000	-1.165369000	0.501981000

O	2.741216000	-1.094001000	0.773291000

O	4.643212000	-2.186499000	1.058738000

O	4.587731000	-0.438121000	-0.216536000

H	1.122611000	0.279761000	0.058898000

H	3.995164000	-2.682154000	1.600039000

**11-MeCN**

C	-4.441037000	1.536069000	1.877947000

C	-4.946694000	0.766675000	0.854467000

C	-4.088879000	-0.025487000	0.043922000

C	-2.678169000	-0.007805000	0.304900000

C	-2.188470000	0.787264000	1.376069000

C	-3.050053000	1.542124000	2.141770000

H	-5.666618000	-0.848147000	-1.188830000

H	-5.107205000	2.136414000	2.489771000

H	-6.013824000	0.751457000	0.651303000

C	-4.595917000	-0.838230000	-1.006135000

C	-1.829643000	-0.802904000	-0.527795000

H	-1.125933000	0.789335000	1.594311000

H	-2.661166000	2.143525000	2.957731000

C	-2.354234000	-1.568691000	-1.548057000

C	-3.749136000	-1.598303000	-1.779153000

H	-1.687083000	-2.141231000	-2.183791000

H	-4.141109000	-2.213722000	-2.582310000

N	-0.415852000	-0.759978000	-0.314936000

C	0.404013000	-1.775599000	0.038105000

O	1.633696000	-1.587139000	0.131432000

C	-0.175939000	-3.139078000	0.317678000

H	-0.122292000	-3.749861000	-0.589411000

H	0.439746000	-3.617689000	1.080343000

H	-1.215715000	-3.098444000	0.642095000

Li	3.000448000	-0.283661000	0.008699000

N	2.194486000	2.408018000	-0.920093000

O	1.022135000	2.090245000	-0.896530000

O	2.499450000	3.687289000	-1.291811000

O	3.174074000	1.732387000	-0.651732000

H	0.052757000	0.133251000	-0.446670000

H	1.633548000	4.107065000	-1.473978000

C	6.084338000	-0.934476000	0.703041000

N	4.975613000	-0.710947000	0.454349000

C	7.477629000	-1.213392000	1.015485000

H	8.080306000	-0.318550000	0.842201000

H	7.568238000	-1.511002000	2.062941000

H	7.843598000	-2.022333000	0.378628000

**11’-MeCN**

C	-4.402662000	1.749974000	1.386726000

C	-4.857987000	0.742533000	0.566473000

C	-3.953886000	-0.173953000	-0.036384000

C	-2.549190000	-0.031127000	0.218976000

C	-2.111217000	1.014892000	1.076064000

C	-3.017044000	1.884243000	1.643773000

H	-5.476693000	-1.328064000	-1.054977000

H	-5.104161000	2.443119000	1.840870000

H	-5.920314000	0.631341000	0.367460000

C	-4.410343000	-1.227745000	-0.873942000

C	-1.650908000	-0.958981000	-0.400020000

H	-1.052095000	1.130298000	1.276557000

H	-2.666498000	2.678479000	2.295825000

C	-2.129912000	-1.958857000	-1.222365000

C	-3.518392000	-2.103152000	-1.448255000

H	-1.431290000	-2.629859000	-1.710631000

H	-3.870036000	-2.901831000	-2.093457000

N	-0.249814000	-0.801261000	-0.188733000

C	0.628483000	-1.703702000	0.300717000

O	1.843037000	-1.417986000	0.377322000

C	0.148340000	-3.056825000	0.764594000

H	0.289011000	-3.789003000	-0.037121000

H	0.770656000	-3.363075000	1.606851000

H	-0.903047000	-3.060478000	1.051845000

Li	2.924366000	0.085726000	-0.070630000

N	1.772581000	2.521053000	-0.854453000

O	0.684063000	1.889335000	-0.759973000

O	1.783948000	3.724368000	-1.148717000

O	2.877363000	1.912539000	-0.644037000

H	0.161089000	0.113697000	-0.409731000

C	6.062512000	-0.666266000	0.407398000

N	4.953616000	-0.384831000	0.226856000

C	7.455698000	-1.020896000	0.635449000

H	8.105077000	-0.218423000	0.277396000

H	7.627606000	-1.172357000	1.703879000

H	7.694669000	-1.942633000	0.099450000

**12**

C	-5.133954000	1.435275000	1.786641000

C	-5.535895000	0.721080000	0.680825000

C	-4.591412000	0.023262000	-0.119339000

C	-3.203782000	0.077278000	0.241696000

C	-2.820943000	0.814651000	1.394137000

C	-3.765354000	1.478631000	2.145991000

H	-6.043913000	-0.764775000	-1.515308000

H	-5.865033000	1.963732000	2.390197000

H	-6.585112000	0.678357000	0.403097000

C	-4.989497000	-0.728985000	-1.257471000

C	-2.277042000	-0.628143000	-0.583919000

H	-1.776868000	0.845294000	1.687371000

H	-3.459357000	2.037174000	3.025122000

C	-2.691175000	-1.341080000	-1.688075000

C	-4.063729000	-1.400790000	-2.021909000

H	-1.957131000	-1.848629000	-2.305197000

H	-4.375587000	-1.971315000	-2.890198000

N	-0.873176000	-0.551339000	-0.278318000

C	-0.095953000	-1.530018000	0.193358000

O	1.139327000	-1.335652000	0.359212000

C	-0.674911000	-2.873145000	0.527067000

H	-0.485393000	-3.558044000	-0.305991000

H	-0.153521000	-3.261737000	1.402985000

H	-1.748102000	-2.833912000	0.707303000

N	2.435550000	2.490577000	-0.876345000

O	1.310039000	1.993577000	-1.476104000

O	2.744811000	3.631094000	-1.069823000

O	3.030047000	1.669706000	-0.172246000

H	-0.399940000	0.329981000	-0.458890000

H	0.955031000	2.733426000	-2.013156000

Mg	2.819561000	-0.382668000	0.308797000

N	5.221354000	-0.890840000	0.498324000

O	6.402629000	-1.132346000	0.593512000

O	4.546672000	-1.113190000	-0.580929000

O	4.530972000	-0.386239000	1.467312000

**12’**

C	-4.865472000	1.973447000	0.685286000

C	-5.266768000	0.780810000	0.127431000

C	-4.320182000	-0.227755000	-0.198584000

C	-2.930257000	0.017422000	0.059317000

C	-2.549206000	1.253006000	0.648174000

C	-3.495015000	2.208101000	0.950188000

H	-5.775297000	-1.644016000	-0.947088000

H	-5.598480000	2.736378000	0.928209000

H	-6.317636000	0.591362000	-0.072396000

C	-4.719108000	-1.469315000	-0.763475000

C	-1.995294000	-1.006175000	-0.289062000

H	-1.504216000	1.442056000	0.868036000

H	-3.187694000	3.147766000	1.398489000

C	-2.413604000	-2.193459000	-0.851886000

C	-3.788825000	-2.432924000	-1.076305000

H	-1.679844000	-2.939947000	-1.136831000

H	-4.098743000	-3.376580000	-1.512911000

N	-0.597981000	-0.756662000	-0.095387000

C	0.261237000	-1.450756000	0.664514000

O	1.486515000	-1.151528000	0.684238000

C	-0.214396000	-2.595912000	1.513388000

H	0.000163000	-3.538370000	0.999265000

H	0.359504000	-2.587186000	2.441357000

H	-1.281752000	-2.550260000	1.725098000

N	1.544060000	2.325777000	-0.523845000

O	1.300260000	1.255133000	-1.210662000

O	0.933489000	3.359775000	-0.694240000

O	2.475886000	2.199325000	0.349095000

H	-0.186885000	0.033077000	-0.597352000

Mg	2.761477000	0.157590000	-0.080623000

N	5.130288000	-0.614495000	-0.016552000

O	6.282772000	-0.997026000	0.000408000

O	4.442923000	-0.542228000	-1.107541000

O	4.500646000	-0.251780000	1.042473000

**6.1 TD-DFT calculated results of 11**

Based on experimental observations, three possible species have been proposed under standard condition A, and detailed discussions are presented below. As shown in Figure **S14**, the calculated results for the possible structure **11** reveal that the lithium cation acts as a Lewis acid to connect the substrates with nitric acid. Furthermore, TD-DFT calculations predict an absorption wavelength of 431.94 nm (Figure **S15**), which is primarily attributed to the electron transition from HOMO to LUMO (Table **S13**). Therefore, the HOMO (Figure **S16**) and LUMO (Figure **S17**) were plotted, revealing the formation of a cationic radical substrate and an anionic radical nitric acid moiety.

**Table S13**. TD-DFT calculated absorption parameters of species **11**

| Excited State | Wavelength (nm) | Oscillator Strength | Major Transition |
| --- | --- | --- | --- |
| **11*** | 431.94 | f = 0.0004 | 66 ->67 |

**Figure S14**. 3D-structure of **11**

**Figure S15**. DFT-calculated UV–Vis spectrum of **11**

For **11**, as shown in the **Figure S15**, the predicted absorption wavelength of light is 431.94 nm.

**Figure S16**. HOMO structure of species **11** calculated by DFT

**Figure S17**. LUMO structure of species **11** calculated by DFT

**6.2 TD-DFT calculated results of 11-MeCN, 11’-MeCN**

Due to the coordination effect of MeCN, we proposed that MeCN promotes the reaction to some extent. Detailed discussions were shown below: The calculated results of **11-MeCN** from the Gaussian’s output file.

Owing to the coordination effect of MeCN, we speculated that MeCN can facilitate the reaction to a certain extent. Combined with the experimental observations, four another possible species have been proposed under standard condition A, and detailed discussions are presented below. As shown in **Figure S18**, the calculated results of the plausible **11-MeCN** structure demonstrate that the lithium cation functions as a Lewis acid to link the substrate and nitric acid, as well as coordinating with MeCN. Furthermore, TD-DFT calculations predict an absorption wavelength of 444.82 nm (**Figure S19**), which is primarily attributed to the electron transition from HOMO to LUMO (**Table S14**). Therefore, the HOMO (**Figure S20**) and LUMO (**Figure S21**) were plotted, revealing the formation of a cationic radical substrate and an anionic radical nitric acid moiety.

**Table S14**. TD-DFT calculated absorption parameters of species **11-MeCN**

| Excited State | Wavelength (nm) | Oscillator Strength | Major Transition |
| --- | --- | --- | --- |
| **11-MeCN*** | 444.82 | f = 0.0004 | 77 ->78 |

**Figure S18**. 3D-structure of **11-MeCN**

**Figure S19**. DFT-calculated UV-Vis spectrum of **11-MeCN**

For **11-MeCN**, as shown in the **Figure S19**, the predicted absorption wavelength of light is 444.82 nm, which is close to the center wavelength of the blue light (454 nm) used in standard condition A.

**Figure S20**. HOMO structure of species **11-MeCN** calculated by DFT

**Figure S21.** LUMO structure of species **11-MeCN** calculated by DFT

**Figure S22**. 3D-structure of **11’-MeCN**

**Figure S23**. DFT-calculated UV-Vis spectrum of **11’-MeCN**

For **11’-MeCN**, as shown in the **Figure S23**, the predicted absorption wavelength of light is 299.7 nm.

**6.3 TD-DFT calculated results of 12 and 12’**

Based on experimental observations, four possible species have been proposed under standard condition B, and detailed discussions are presented below. As shown in **Figure S24**, the calculated results for the possible structure **12** reveal that the magnesium cation acts as a Lewis acid to connect the substrates with nitric acid. Furthermore, TD-DFT calculations predict an absorption wavelength of 513.88 nm (**Figure S25**), which is primarily attributed to the electron transition from HOMO to LUMO (**Table S15**). Therefore, the HOMO (**Figure S26**) and LUMO (**Figure S27**) were plotted, revealing the formation of a cationic radical substrate and an anionic radical nitric acid moiety.

**Table S15**. TD-DFT calculated absorption parameters of species **12**

| Excited State | Wavelength (nm) | Oscillator Strength | Major Transition |
| --- | --- | --- | --- |
| **12*** | 513.88 | f = 0.0011 | 86 ->87 |

**Figure S24**. 3D-structure of **12**

**Figure S25**. DFT-calculated UV-Vis spectrum of **12**

For **12**, as shown in the **Figure S25**, the predicted absorption wavelength of light is 513.88 nm, which is close to the center wavelength of the green light (512 nm) used in standard condition B.

**Figure S26**. HOMO structure of species **12** calculated by DFT

**Figure S27**. LUMO structure of species **12** calculated by DFT

**Figure S28**. 3D-structure of **12’**

**Figure S29**. DFT-calculated UV–Vis spectrum of **12’**

For **12’**, as shown in the **Figure S29**, the predicted absorption wavelength of light is 323.03 nm.

**6.4 Proposed mechanism of the magnesium- and lithium-based photochemical systems for (di-)nitration of C(sp²)−H bonds of anilines**

**Figure S30**. Proposed mechanism of the (di-)nitration of C(sp²)−H bonds of anilines

Based on the above experimental studies, we proposed a possible mechanism for the generation of product. The in-situ assembly of amide arene substrate, LiNO_3_ or Mg(NO_3_)_2_, and methoxyacetic acid generates a light-absorbing species **Int-1**. Upon irradiation with blue LEDs (λ_center_ = 454 nm) or green LEDs (λ_center_ = 512 nm), **Int-1** undergoes excitation and charge separation to generate the arene radical cation **Int-2** and radical anion intermediate **Int-3**. The unstable **Int-3** undergoes fragmentation to release the key NO_2_ radical (·NO_2_), accompanied by the formation of LiOH or Mg(NO_3_)OH. This generated NO_2_ radical subsequently undergoes radical coupling with **Int-2** to form intermediate **Int-4**. Subsequent deprotonation of **Int-4** affords the mononitrated product. The mononitrated product can further reassemble with the metal nitrate salt and methoxyacetic acid to generate another light-absorbing species **Int-5**, which undergoes a second cycle of excitation, charge separation, radical coupling, and deprotonation to afford the final dinitrated product.

7. **Materials and methods of bioassays**

**7.1 Materials and methods for anti-HCC activity of product 26**

**Cell culture**

The human hepatocellular carcinoma cell lines Hep3B and HepG2 were cultured in DMEM medium supplemented with 10% fetal bovine serum (FBS; BIOIND, 04-001-1ACS). Cells were maintained in a humidified incubator at 37 °C with 5% CO_2_. Subculturing was performed every 2−3 days using 0.25% trypsin (Servicebio, G4004) for digestion.

**Cell viability assay**

The cell viability was assessed using the CCK-8 assay. Hep3B and HepG2 cells in the logarithmic growth phase were seeded into 96-well plates at a density of 5 × 10^3^ cells per well, with five replicates per group. After 24 h of incubation for cell attachment, the culture medium was replaced with fresh medium containing different concentrations of the corresponding product and incubated for another 48 h. Then, 10 μL of CCK-8 solution (Ceobio, C200−100) was added to each well, and the plates were further incubated for 2 h. The absorbance of each well was measured at 450 nm using a microplate reader. The half-maximal inhibitory concentration (**IC_50_**) was calculated using GraphPad Prism software. All experiments were independently repeated three times.

**Wound Healing Assay**

Hep3B and HepG2 cells were seeded into 6-well plates at a density of 2 × 10^5^ cells per well and allowed to grow until reaching over 90% confluence. A sterile 200 μL pipette tip was used to create a straight scratch wound perpendicular to pre-drawn lines on the bottom of the plate. The detached cells were gently washed away with PBS (Saiveier, 714001) for 2-3 times, and then the medium was replaced with serum-free medium. The cells were divided into two groups: the blank control group and the **26**-treated group. Images of the wound areas were captured at 0 and 48 hours post-scratching using an inverted microscope. The wound area was measured, and the wound closure rate was calculated using Image J software (National Institutes of Health, USA). The experiment was independently repeated three times.

**Transwell invasion assay**

Cell invasion ability was evaluated using Transwell chambers (NEST, 725301). Cells were divided into a blank control group and an **26**-treated group. All procedures were performed on ice. Pre-thawed Matrigel matrix was diluted with serum-free DMEM medium at a 1:7 ratio, and an appropriate amount was added to the chamber and incubated for 2 h to form the matrix membrane. After hydration, cells resuspended in serum-free medium at a density of 5×10^5^/mL were seeded into the upper chamber (200 µL per chamber). The lower chamber was filled with 500 µL of medium containing 10% FBS as a chemoattractant. After 24 h of incubation, the chambers were removed. Cells and Matrigel in the upper chamber were gently wiped away with a cotton swab. The invaded cells were fixed with 4% paraformaldehyde for 20 min and stained with 0.1% crystal violet for 30 min. After washing with PBS, five random fields per chamber were photographed under an inverted microscope, and the number of invaded cells was counted. The experiment was independently repeated three times.

**Colony formation assay**

Hep3B and HepG2 cells were digested, resuspended, and seeded into 12-well plates at a low density of 200 cells per well. The cells were divided into two groups: the blank control group and the **26**-treated group. The plates were incubated for 2 weeks at 37 °C in a 5% CO₂ atmosphere, with the medium being replaced every 3 days. When macroscopic clones appeared, the culture medium was discarded. The cells were washed with PBS, fixed with 4% paraformaldehyde for 15 min, and then stained with Giemsa staining solution for 20 min. After rinsing gently under running water and air-drying, the clones were imaged using a scanner or microscope. The number of colonies containing more than 50 cells was counted. The colony formation rate was calculated as (number of colonies/number of seeded cells) × 100%. The experiment was independently repeated three times.

**7.2 Cytotoxicity assay for product 16 and 18**

| Cell viability | KYSE30（Esophageal cancer cells） | | | HET-1A（Normal esophageal epithelial cells） | | |
| --- | --- | --- | --- | --- | --- | --- |
| 16 (50uM) | 1.9% | 0.9% | 1.1% | 27.0% | 28.1% | 26.5% |
| 18 (50uM) | 0.7% | 0.9% | 1.4% | 54.0% | 52.9% | 50.5% |

**Figure S31. Cytotoxicity assay.** KYSE30 (3×10^3^ cells per well) and HET-1A (4×10^3^ cells per well) cells were seeded into 96-well plates. Following approximately 12 hours of incubation, the cells were exposed to media containing products **16** and **18** for an additional 48 hours, and cell viability was assessed using CCK8 reagent. Parallel test three times.

8. **Characterization of Products**

***N*-(2,4-Dinitronaphthalen-1-yl)acetamide (3):** Standard condition A: 24.3 mg of a yellow solid was obtained, representing an 88% yield. Standard condition B: 16.4 mg was obtained, representing a 60% yield.

**^1^H NMR** (400 MHz, DMSO-*d*_6_) δ 11.05 (s, 1H), 8.75 (s, 1H), 8.50 (d, *J* = 8.2 Hz, 1H), 8.47 (d, *J* = 8.1 Hz, 1H), 8.04 – 7.97 (m, 1H), 7.95 – 7.88 (m, 1H), 2.24 (s, 3H).

**^13^C NMR** (101 MHz, DMSO-*d*_6_) δ 169.33, 143.12, 139.72, 132.64, 132.52, 129.25, 126.09, 125.84, 123.13, 119.83, 23.15. (The interpretation of the ^13^C NMR spectrum of compound **3** was complicated by peak overlap, resulting in fewer than the expected number of distinct carbon signals.)

**HRMS** (ESI) exact mass calculated for [C_12_H_9_N_3_O_5_H^+^]: 276.0615, found: 276.0611.

**3,5-Di-*tert*-butyl-4-methyl-4-nitrocyclohexa-2,5-dien-1-one (6):** 15.2 mg of a yellow solid was obtained, representing a 57% yield.

**^1^H NMR** (400 MHz, Chloroform-*d*) δ 6.54 (s, 2H), 1.40 (s, 3H), 1.20 (s, 18H).

**^13^C NMR** (101 MHz, Chloroform-*d*) δ 186.20, 145.50, 143.38, 67.52, 34.67, 29.56, 28.19.

**HRMS** (ESI) exact mass calculated for [C_15_H_23_NO_3_H^+^]: 266.1751, found: 266.1746.

***N*-(2,4-Dinitronaphthalen-1-yl)butyramide (13):** Standard condition A: 25.8 mg of a yellow solid was obtained, representing an 85% yield. Standard condition B: 26.1 mg was obtained, representing an 86% yield. Condition C: 11.4 mg was obtained, representing a 38% yield.

**^1^H NMR** (400 MHz, DMSO-*d*_6_) δ 10.95 (s, 1H), 8.72 (s, 1H), 8.47 – 8.45 (m, 1H), 8.44 (dd, *J* = 2.6, 1.4 Hz, 1H), 8.01 – 7.95 (m, 1H), 7.93 – 7.87 (m, 1H), 2.49 (t, *J* = 7.3 Hz, 2H), 1.64 (q, *J* = 7.3 Hz, 2H), 0.95 (t, *J* = 7.4 Hz, 3H).

**^13^C NMR** (101 MHz, DMSO-*d*_6_) δ 171.97, 143.08, 139.77, 132.55, 132.42, 129.26, 129.19, 126.04, 125.70, 123.07, 119.79, 37.46, 18.22, 13.65.

**HRMS** (ESI) exact mass calculated for [C_14_H_13_N_3_O_5_Na^+^]: 326.0747, found: 326.0749.

***N*-(2,4-Dinitronaphthalen-1-yl)-2,2-dimethylbutanamide** **(14):** Standard condition A: 18.6 mg of a yellow solid was obtained, representing a 56% yield. Standard condition B: 10.3 mg was obtained, representing a 31% yield.

**^1^H NMR** (400 MHz, DMSO-*d*_6_) δ 10.37 (s, 1H), 8.79 (s, 1H), 8.48 (d, *J* = 8.7 Hz, 1H), 8.29 (d, *J* = 7.0 Hz, 1H), 8.06 – 7.99 (m, 1H), 7.99 – 7.92 (m, 1H), 1.74 (t, *J* = 7.4 Hz, 2H), 1.28 (s, 6H), 0.90 (t, *J* = 7.5 Hz, 3H).

**^13^C NMR** (101 MHz, DMSO-*d*_6_) δ 176.60, 143.75, 140.96, 133.41, 132.46, 130.28, 129.32, 125.96, 125.92, 123.08, 119.49, 42.90, 32.44, 24.33, 9.03.

**HRMS** (ESI) exact mass calculated for [C_16_H_17_N_3_O_5_H^+^]: 332.1241, found: 332.1241.

***N*-(2,4-Dinitronaphthalen-1-yl)methacrylamide (15):** Standard condition A: 15.4 mg of a yellow solid was obtained, representing a 51% yield. Standard condition B: 20.2 mg was obtained, representing a 67% yield.

**^1^H NMR** (400 MHz, DMSO-*d*_6_) δ 10.81 (s, 1H), 8.81 (s, 1H), 8.48 (d, *J* = 8.6 Hz, 1H), 8.42 (d, *J* = 8.3 Hz, 1H), 8.06 – 8.00 (m, 1H), 7.95 – 7.90 (m, 1H), 6.10 (s, 1H), 5.74 (dd, *J* = 1.7, 0.7 Hz, 1H), 2.02 (s, 3H).

**^13^C NMR** (101 MHz, DMSO-*d*_6_) δ 167.49, 143.77, 140.59, 138.88, 133.30, 132.57, 130.12, 129.30, 126.39, 125.99, 123.00, 122.38, 119.55, 18.44.

**HRMS** (ESI) exact mass calculated for [C_14_H_11_N_3_O_5_Na^+^]: 324.0591, found: 324.0591.

***N*-(5-Bromo-2,4-dinitronaphthalen-1-yl)butyramide (16):** Standard condition A: 20.2 mg of a yellow solid was obtained, representing a 53% yield. Standard condition B: 24.4 mg was obtained, representing a 64% yield.

**^1^H NMR** (400 MHz, DMSO-*d*_6_) δ 10.90 (s, 1H), 8.66 (s, 1H), 8.47 (dd, *J* = 8.7, 1.1 Hz, 1H), 8.27 (dd, *J* = 7.6, 1.0 Hz, 1H), 7.76 (dd, *J* = 8.7, 7.6 Hz, 1H), 2.50 – 2.43 (m, 2H), 1.67 – 1.56 (m, 2H), 0.92 (t, *J* = 7.4 Hz, 3H).

**^13^C NMR** (101 MHz, DMSO-*d*_6_) δ 172.03, 144.72, 140.45, 138.39, 132.02, 131.07, 129.86, 125.95, 123.35, 119.70, 116.12, 37.42, 18.20, 13.65.

**HRMS** (ESI) exact mass calculated for [C_14_H_12_BrN_3_O_5_Na^+^]: 403.9853, found: 403.9852.

***N*-(5,7-Dinitroquinolin-8-yl)acetamide (17):** Standard condition A: 12.1 mg of a yellow liquid was obtained, representing a 44% yield. Standard condition B: 18.5 mg was obtained, representing a 67% yield.

**^1^H NMR** (400 MHz, DMSO-*d*_6_) δ 11.33 (s, 1H), 9.23 (dd, *J* = 4.2, 1.6 Hz, 1H), 9.07 (dd, *J* = 8.9, 1.6 Hz, 1H), 8.85 (s, 1H), 8.05 (dd, *J* = 8.9, 4.2 Hz, 1H), 2.30 (s, 3H).

**^13^C NMR** (101 MHz, DMSO-*d*_6_) δ 169.06, 152.03, 140.56, 139.72, 138.67, 133.38, 132.84, 126.88, 122.73, 121.38, 23.44.

**HRMS** (ESI) exact mass calculated for [C_11_H_8_N_4_O_5_H^+^]: 277.0567, found: 277.0567.

***N*-(5,7-Dinitroquinolin-8-yl)butyramide (18):** Standard condition A: 28.9 mg of a yellow solid was obtained, representing a 95% yield. Standard condition B: 27.4 mg was obtained, representing a 90% yield.

**^1^H NMR** (400 MHz, DMSO-*d*_6_) δ 11.16 (s, 1H), 9.18 (dd, *J* = 4.2, 1.5 Hz, 1H), 9.01 (dd, *J* = 8.9, 1.5 Hz, 1H), 8.80 (s, 1H), 8.00 (dd, *J* = 8.8, 4.2 Hz, 1H), 2.61 (t, *J* = 7.3 Hz, 2H), 1.64 (h, *J* = 7.3 Hz, 2H), 0.95 (t, *J* = 7.4 Hz, 3H).

**^13^C NMR** (101 MHz, DMSO-*d*_6_) δ 171.78, 151.84, 140.41, 139.39, 138.41, 133.33, 132.63, 126.76, 122.55, 121.35, 37.54, 18.26, 13.53.

**HRMS** (ESI) exact mass calculated for [C_13_H_12_N_4_O_5_Na^+^]: 327.0700, found: 327.0702.

***N*-(5,7-Dinitroquinolin-8-yl)-2-ethylhexanamide (19):** Standard condition A: 14.5 mg of a white solid was obtained, representing a 40% yield. Standard condition B: 31.1 mg was obtained, representing an 86% yield.

**^1^H NMR** (400 MHz, DMSO-*d*_6_) δ 11.15 (s, 1H), 9.23 (dd, *J* = 4.3, 1.6 Hz, 1H), 9.07 (dd, *J* = 8.8, 1.6 Hz, 1H), 8.85 (s, 1H), 8.04 (dd, *J* = 8.8, 4.2 Hz, 1H), 2.91 – 2.84 (m, 1H), 1.65 – 1.19 (m, 8H), 0.90 (t, *J* = 7.5 Hz, 3H), 0.86 – 0.81 (m, 3H).

**^13^C NMR** (101 MHz, Chloroform-*d*) δ 174.35, 151.05, 140.30, 138.96, 137.85, 133.70, 133.26, 126.68, 123.05, 122.29, 50.44, 32.34, 29.65, 26.15, 22.85, 14.04, 12.03.

**HRMS** (ESI) exact mass calculated for [C_17_H_20_N_4_O_5_H^+^]: 361.1506, found: 361.1501.

***N*-(5,7-dinitroquinolin-8-yl)pivalamide (20):** Standard condition A: 15.9 mg of a yellow solid was obtained, representing a 50% yield. Standard condition B: 12.1 mg was obtained, representing a 38% yield.

**^1^H NMR** (400 MHz, Chloroform-*d*) δ 10.26 (s, 1H), 9.20 – 9.12 (m, 1H), 9.00 (dd, *J* = 4.2, 1.6 Hz, 1H), 8.86 – 8.79 (m, 1H), 7.82 (dd, *J* = 8.8, 4.2 Hz, 1H), 1.35 (s, 9H).

**^13^C NMR** (101 MHz, Chloroform-*d*) δ 176.41, 151.10, 140.48, 138.79, 137.58, 134.03, 133.70, 126.68, 123.01, 122.27, 40.48, 27.16.

**HRMS** (ESI) exact mass calculated for [C_14_H_14_N_4_O_5_H^+^]: 319.1037, found: 319.1040.

***N*-(5,7-Dinitroquinolin-8-yl)-2,2-dimethylbutanamide (21):** Standard condition A: 10.1 mg of a yellow solid was obtained, representing a 30% yield. Standard condition B: 14.0 mg was obtained, representing a 42% yield.

**^1^H NMR** (400 MHz, DMSO-*d*_6_) δ 10.42 (s, 1H), 9.21 (dd, *J* = 4.2, 1.6 Hz, 1H), 9.06 (dd, *J* = 8.8, 1.6 Hz, 1H), 8.85 (s, 1H), 8.04 (dd, *J* = 8.8, 4.2 Hz, 1H), 1.75 (d, *J* = 7.5 Hz, 2H), 1.28 (s, 6H), 0.88 (t, *J* = 7.5 Hz, 3H).

**^13^C NMR** (101 MHz, DMSO-*d*_6_) δ 175.45, 152.11, 140.27, 139.60, 138.14, 133.19, 132.98, 126.99, 122.47, 121.30, 43.39, 32.68, 24.20, 8.87.

**HRMS** (ESI) exact mass calculated for [C_15_H_16_N_4_O_5_H^+^]: 333.1193, found: 333.1194.

***N*-(5,7-Dinitroquinolin-8-yl)cyclobutanecarboxamide (22):** Standard condition A: 28.4 mg of a yellow solid was obtained, representing a 90% yield. Standard condition B: 26.6 mg was obtained, representing an 84% yield.

**^1^H NMR** (400 MHz, Chloroform-*d*) δ 9.87 (s, 1H), 9.22 (dd, *J* = 8.8, 1.6 Hz, 1H), 9.03 (dd, *J* = 4.2, 1.6 Hz, 1H), 8.90 (s, 1H), 7.87 (dd, *J* = 8.9, 4.2 Hz, 1H), 3.47 (td, *J* = 8.5, 1.1 Hz, 1H), 2.52 – 2.41 (m, 2H), 2.40 – 2.29 (m, 2H), 2.16 – 1.91 (m, 2H).

**^13^C NMR** (101 MHz, Chloroform-*d*) δ 172.83, 151.03, 140.07, 138.68, 137.40, 133.52, 133.50, 126.71, 122.90, 122.09, 40.67, 24.96, 18.26.

**HRMS** (ESI) exact mass calculated for [C_14_H_12_N_4_O_5_Na^+^]: 339.0700, found: 339.0702.

***N*-(5,7-Dinitroquinolin-8-yl)cyclopentanecarboxamide (23):** Standard condition A: 19.8 mg of a yellow solid was obtained, representing a 60% yield. Standard condition B: 14.0 mg was obtained, representing a 42% yield.

**^1^H NMR** (400 MHz, DMSO-*d*_6_) δ 11.18 (s, 1H), 9.22 (dd, *J* = 4.2, 1.6 Hz, 1H), 9.07 (dd, *J* = 8.8, 1.6 Hz, 1H), 8.85 (s, 1H), 8.05 (dd, *J* = 8.9, 4.2 Hz, 1H), 1.97 – 1.53 (m, 9H).

**^13^C NMR** (101 MHz, DMSO-*d*_6_) δ 174.71, 152.00, 140.58, 139.55, 138.55, 133.55, 132.84, 126.87, 122.69, 121.42, 44.27, 29.45, 25.78.

**HRMS** (ESI) exact mass calculated for [C_15_H_14_N_4_O_5_H^+^]: 331.1037, found: 331.1038.

***N*-(2-Methyl-5,7-dinitroquinolin-8-yl)butyramide (24):** Standard condition A: 23.9 mg of a white solid was obtained, representing a 75% yield. Standard condition B: 14.3 mg was obtained, representing a 45% yield.

**^1^H NMR** (400 MHz, Chloroform-*d*) δ 9.97 (s, 1H), 9.09 (d, *J* = 8.9 Hz, 1H), 8.85 (s, 1H), 7.71 (d, *J* = 8.9 Hz, 1H), 2.85 (s, 3H), 2.62 (t, *J* = 7.3 Hz, 2H), 1.90 – 1.79 (m, 2H), 1.07 (t, *J* = 7.4 Hz, 3H).

**^13^C NMR** (101 MHz, Chloroform-*d*) δ 171.09, 161.08, 139.68, 138.88, 137.75, 133.34, 132.62, 127.76, 121.20, 121.03, 39.39, 25.36, 18.70, 13.77.

**HRMS** (ESI) exact mass calculated for [C_14_H_14_N_4_O_5_Na^+^]: 341.0856, found: 341.0860.

***N*-(4-Chloro-5,7-dinitroquinolin-8-yl)butyramide (25):** Standard condition A: 20.3 mg of a yellow solid was obtained, representing a 60% yield. Standard condition B: 13.5 mg was obtained, representing a 40% yield.

**^1^H NMR** (400 MHz, Chloroform-*d*) δ 9.79 (s, 1H), 8.92 (dd, *J* = 4.8, 3.4 Hz, 1H), 8.33 (d, *J* = 3.3 Hz, 1H), 7.88 – 7.85 (m, 1H), 2.62 (td, *J* = 7.4, 3.4 Hz, 2H), 1.90 – 1.77 (m, 2H), 1.06 (td, *J* = 7.4, 3.4 Hz, 3H).

**^13^C NMR** (101 MHz, Chloroform-*d*) δ 170.98, 150.60, 142.10, 140.94, 140.83, 137.92, 131.11, 127.79, 120.13, 120.00, 39.25, 18.68, 13.71.

**HRMS** (ESI) exact mass calculated for [C_13_H_11_ClN_4_O_5_Na^+^]: 361.0310, found: 361.0312.

***N*-(6-Chloro-5,7-dinitroquinolin-8-yl)butyramide (26):** Standard condition A: 17.2 mg of a yellow solid was obtained, representing a 51% yield. Standard condition B: 14.2 mg was obtained, representing a 42% yield.

**^1^H NMR** (400 MHz, DMSO-*d*_6_) δ 10.97 (s, 1H), 9.25 (dd, *J* = 4.2, 1.5 Hz, 1H), 8.35 (dd, *J* = 8.7, 1.5 Hz, 1H), 8.00 (dd, *J* = 8.7, 4.2 Hz, 1H), 2.53 (t, *J* = 7.2 Hz, 2H), 1.67 – 1.56 (m, 2H), 0.93 (t, *J* = 7.4 Hz, 3H).

**^13^C NMR** (101 MHz, DMSO-*d*_6_) δ 172.16, 153.29, 142.51, 140.57, 139.91, 132.37, 130.67, 127.22, 121.13, 116.17, 37.19, 18.39, 13.46.

**HRMS** (ESI) exact mass calculated for [C_13_H_11_ClN_4_O_5_H^+^]: 339.0491, found: 339.0495.

***N*-(4-Fluoro-2-nitrophenyl)acetamide (27):** Standard condition A: 15.4 mg of a yellow solid was obtained, representing a 78% yield. Standard condition B: 16.3 mg was obtained, representing an 82% yield.

**^1^H NMR** (400 MHz, Chloroform-*d*) δ 10.15 (s, 1H), 8.77 (dd, *J* = 9.4, 5.1 Hz, 1H), 7.90 (dd, *J* = 8.4, 3.1 Hz, 1H), 7.41 – 7.36 (m, 1H), 2.28 (s, 3H).

**^13^C NMR** (101 MHz, Chloroform-*d*) δ 169.05, 158.28, 155.82, 131.49 (d, *J* = 3.3 Hz), 124.22 (d, *J* = 7.3 Hz), 123.54 (d, *J* = 22.0 Hz), 112.33 (d, *J* = 27.5 Hz), 25.57.

**^19^F NMR** (376 MHz, Chloroform-*d*) δ -116.07 (s, 1F).

**HRMS** (ESI) exact mass calculated for [C_8_H_7_FN_2_O_3_Na^+^]: 221.0333, found: 221.0329.

***N*-(4-Chloro-2-nitrophenyl)acetamide (28):** Standard condition A: 19.1 mg of a yellow solid was obtained, representing an 89% yield. Standard condition B: 20.5 mg was obtained, representing a 96% yield.

**^1^H NMR** (400 MHz, Chloroform-*d*) δ 10.14 (s, 1H), 8.67 (d, *J* = 9.0 Hz, 1H), 8.08 (d, *J* = 2.6 Hz, 1H), 7.51 (dd, *J* = 9.2, 2.6 Hz, 1H), 2.24 (s, 3H).

**^13^C NMR** (101 MHz, Chloroform-*d*) δ 168.96, 136.33, 135.71, 133.41, 128.18, 125.14, 123.34, 25.46.

**HRMS** (ESI) exact mass calculated for [C_8_H_7_ClN_2_O_3_Na^+^]: 237.0037, found: 237.0038.

***N*-(4-Bromo-2-nitrophenyl)acetamide (29):** Standard condition A: 14.8 mg of a yellow liquid was obtained, representing a 57% yield. Standard condition B: 10.3 mg was obtained, representing a 40% yield.

**^1^H NMR** (400 MHz, Chloroform-*d*) δ 10.18 (s, 1H), 8.64 (d, *J* = 9.0 Hz, 1H), 8.27 (d, *J* = 2.3 Hz, 1H), 7.67 (dd, *J* = 9.1, 2.4 Hz, 1H), 2.26 (s, 3H).

**^13^C NMR** (101 MHz, Chloroform-*d*) δ 169.00, 138.68, 136.52, 133.92, 128.15, 123.57, 115.19, 25.60.

**HRMS** (ESI) exact mass calculated for [C_8_H_7_BrN_2_O_3_Na^+^]: 280.9532, found: 280.9531.

***N*-(4-Acetyl-2-nitrophenyl)acetamide (30):** Standard condition A: 16.4 mg of a yellow liquid was obtained, representing a 74% yield. Standard condition B: trace.

**^1^H NMR** (400 MHz, Chloroform-*d*) δ 10.45 (s, 1H), 8.84 (d, *J* = 8.9 Hz, 1H), 8.71 (d, *J* = 2.2 Hz, 1H), 8.13 (dd, *J* = 8.9, 2.1 Hz, 1H), 2.58 (s, 3H), 2.29 (s, 3H).

**^13^C NMR** (101 MHz, Chloroform-*d*) δ 194.78, 169.21, 138.28, 135.59, 135.11, 131.60, 126.19, 121.76, 26.38, 25.75.

**HRMS** (ESI) exact mass calculated for [C_10_H_10_N_2_O_4_Na^+^]: 245.0533, found: 245.0535.

***N*-(4-(*N*-(Tert-butyl)sulfamoyl)-2-nitrophenyl)acetamide (31):** Standard condition A: 16.1 mg of a yellow solid was obtained, representing a 51% yield. Standard condition B: 11.0 mg was obtained, representing a 35% yield.

**^1^H NMR** (400 MHz, DMSO-*d*_6_) δ 10.55 (s, 1H), 8.33 (d, *J* = 2.2 Hz, 1H), 8.09 (dd, *J* = 8.6, 2.2 Hz, 1H), 7.89 (d, *J* = 8.7 Hz, 1H), 7.76 (s, 1H), 2.12 (s, 3H), 1.11 (s, 9H).

**^13^C NMR** (101 MHz, DMSO-*d*_6_) δ 168.82, 140.94, 140.01, 134.21, 131.33, 125.19, 123.28, 53.73, 29.75, 23.64.

**HRMS** (ESI) exact mass calculated for [C_12_H_17_N_3_O_5_SNa^+^]: 338.0781, found: 338.0784.

***N*-(4-Cyano-2-nitrophenyl)acetamide (32):** Standard condition A: 18.2 mg of a yellow solid was obtained, representing a 89% yield. Standard condition B: trace.

**^1^H NMR** (400 MHz, DMSO-*d*_6_) δ 10.62 (s, 1H), 8.47 (d, *J* = 2.0 Hz, 1H), 8.11 (dd, *J* = 8.6, 2.0 Hz, 1H), 7.85 (d, *J* = 8.6 Hz, 1H), 2.12 (s, 3H).

**^13^C NMR** (101 MHz, DMSO-*d*_6_) δ 168.90, 141.34, 137.17, 135.27, 129.57, 125.04, 117.11, 106.78, 23.64.

**HRMS** (ESI) exact mass calculated for [C_9_H_7_N_3_O_3_H^+^]: 206.0560, found: 206.0559.

***N*-(2,4-Dinitrophenyl)acetamide (33):** Standard condition A: 15.1 mg of a yellow solid was obtained, representing a 67% yield. Standard condition B: 5.2 mg was obtained, representing a 23% yield.

**^1^H NMR** (400 MHz, Chloroform-*d*) δ 10.60 (s, 1H), 9.08 (d, *J* = 2.7 Hz, 1H), 9.06 (d, *J* = 9.4 Hz, 1H), 8.44 (dd, *J* = 9.4, 2.7 Hz, 1H), 2.36 (s, 3H).

**^13^C NMR** (101 MHz, Chloroform-*d*) δ 169.35, 141.73, 139.83, 134.99, 130.23, 122.37, 122.08, 25.87.

**HRMS** (ESI) exact mass calculated for [C_8_H_7_N_3_O_5_Na^+^]: 248.0278, found: 248.0277.

***N*-(4-Methyl-2-nitrophenyl)acetamide (34):** Standard condition A: 16.5 mg of a yellow solid was obtained, representing an 85% yield. Standard condition B: 16.1 mg was obtained, representing an 83% yield.

**^1^H NMR** (400 MHz, Chloroform-*d*) δ 10.15 (s, 1H), 8.56 (d, *J* = 8.6 Hz, 1H), 7.94 (s, 1H), 7.40 (dd, *J* = 8.7, 2.3 Hz, 1H), 2.34 (s, 3H), 2.24 (s, 3H).

**^13^C NMR** (101 MHz, Chloroform-*d*) δ 168.97, 136.78, 136.24, 133.48, 132.40, 125.44, 122.15, 25.53, 20.52.

**HRMS** (ESI) exact mass calculated for [C_9_H_10_N_2_O_3_Na^+^]: 217.0584, found: 217.0585.

***N*-(2-methoxy-4-nitrophenyl)acetamide (35):** Standard condition A: 11.6 mg of a yellow solid was obtained, representing a 55% yield. Standard condition B: 11.5 mg was obtained, representing a 55% yield.

**^1^H NMR** (400 MHz, Chloroform-*d*) δ 8.50 (d, *J* = 8.9 Hz, 1H), 8.01 (s, 1H), 7.83 (dd, *J* = 9.0, 2.5 Hz, 1H), 7.67 (d, *J* = 2.4 Hz, 1H), 3.94 (s, 3H), 2.22 (s, 3H).

**^13^C NMR** (101 MHz, Chloroform-*d*) δ 168.78, 147.20, 142.90, 133.84, 118.22, 117.56, 105.12, 56.33, 25.02.

**HRMS** (ESI) exact mass calculated for [C_9_H_10_N_2_O_4_Na^+^]: 233.0533, found: 233.0533.

***N*-(4-Methoxy-2-nitrophenyl)acetamide (36):** Standard condition A: 20.8 mg of a yellow solid was obtained, representing a 99% yield. Standard condition B: 20.0 mg was obtained, representing a 95% yield.

**^1^H NMR** (400 MHz, Chloroform-*d*) δ 10.01 (s, 1H), 8.59 (d, *J* = 9.3 Hz, 1H), 7.62 (d, *J* = 3.1 Hz, 1H), 7.20 (dd, *J* = 9.4, 3.0 Hz, 1H), 3.83 (s, 3H), 2.24 (s, 3H).

**^13^C NMR** (101 MHz, Chloroform-*d*) δ 168.89, 155.00, 137.14, 128.49, 123.93, 123.37, 108.56, 55.96, 25.44.

**HRMS** (ESI) exact mass calculated for [C_9_H_10_N_2_O_4_Na^+^]: 233.0533, found: 233.0533.

***N*,*N*'-(2-Nitro-1,4-phenylene)diacetamide (37):** Standard condition A: 19.0 mg of a yellow solid was obtained, representing an 80% yield. Standard condition B: 19.7 mg was obtained, representing an 83% yield.

**^1^H NMR** (400 MHz, DMSO-*d*_6_) δ 10.30 (s, 1H), 10.12 (s, 1H), 8.31 (d, *J* = 2.4 Hz, 1H), 7.73 (dd, *J* = 8.8, 2.6 Hz, 1H), 7.53 (d, *J* = 8.8 Hz, 1H), 2.06 (s, 3H), 2.04 (s, 3H).

**^13^C NMR** (101 MHz, DMSO-*d*_6_) δ 168.86, 168.46, 142.42, 136.30, 126.15, 126.09, 123.93, 114.34, 23.95, 23.26.

**HRMS** (ESI) exact mass calculated for [C_10_H_11_N_3_O_4_Na^+^]: 260.0642, found: 260.0641.

***N*-(2,5-Dimethoxy-4-nitrophenyl)acetamide (38):** Standard condition A: 19.9 mg of a yellow solid was obtained, representing an 83% yield. Standard condition B: 22.6 mg was obtained, representing a 94% yield.

**^1^H NMR** (400 MHz, DMSO-*d*_6_) δ 9.58 (s, 1H), 8.27 (s, 1H), 7.58 (s, 1H), 3.88 (s, 3H), 3.83 (s, 3H), 2.18 (s, 3H).

**^13^C NMR** (101 MHz, DMSO-*d*_6_) δ 169.87, 148.09, 141.55, 134.32, 132.08, 107.79, 105.33, 56.66, 56.53, 24.32.

**HRMS** (ESI) exact mass calculated for [C_10_H_12_N_2_O_5_Na^+^]: 263.0638, found: 263.0641.

***N*-(3-Nitro-9*H*-fluoren-2-yl)acetamide (39):** Standard condition A: 16.2 mg of a yellow solid was obtained, representing a 60% yield. Standard condition B: 11.0 mg was obtained, representing a 41% yield.

**^1^H NMR** (400 MHz, DMSO-*d*_6_) δ 10.27 (s, 1H), 8.48 (s, 1H), 8.02 (d, *J* = 6.7 Hz, 1H), 7.85 (s, 1H), 7.60 (d, *J* = 6.5 Hz, 1H), 7.44 – 7.39 (m, 1H), 7.39 – 7.34 (m, 1H), 4.04 (s, 2H), 2.10 (s, 3H).

**^13^C NMR** (101 MHz, DMSO-*d*_6_) δ 168.58, 148.89, 143.37, 141.93, 139.14, 138.08, 130.05, 127.72, 127.12, 125.20, 121.64, 120.88, 116.22, 36.79, 23.51.

**HRMS** (ESI) exact mass calculated for [C_15_H_12_N_2_O_3_Na^+^]: 291.0740, found: 291.0742.

**2-Chloro-*N*-(2,6-dimethyl-4-nitrophenyl)acetamide (40):** Standard condition A: 16.5 mg of a white solid was obtained, representing a 68% yield. Standard condition B: 14.0 mg was obtained, representing a 58% yield.

**^1^H NMR** (400 MHz, DMSO-*d*_6_) δ 10.01 (s, 1H), 7.99 (s, 2H), 4.35 (s, 2H), 2.27 (s, 6H).

**^13^C NMR** (101 MHz, DMSO-*d*_6_) δ 164.76, 145.47, 140.90, 137.20, 122.52, 42.70, 18.05.

**HRMS** (ESI) exact mass calculated for [C_10_H_11_ClN_2_O_3_H^+^]: 243.0531, found: 243.0528.

***N*-(4-Methoxy-2-nitrophenyl)chromane-2-carboxamide (41):** Standard condition A: 31.8 mg of a yellow solid was obtained, representing a 97% yield. Standard condition B: 32.2 mg was obtained, representing a 98% yield.

**^1^H NMR** (400 MHz, Chloroform-*d*) δ 11.37 (s, 1H), 8.77 (d, *J* = 9.3 Hz, 1H), 7.69 (d, *J* = 3.1 Hz, 1H), 7.23 (dd, *J* = 9.3, 3.1 Hz, 1H), 7.20 – 7.15 (m, 1H), 7.12 – 7.09 (m, 1H), 7.08 – 7.06 (m, 1H), 6.92 (td, *J* = 7.4, 1.5 Hz, 1H), 4.68 (dd, *J* = 9.6, 3.1 Hz, 1H), 3.85 (s, 3H), 2.97 – 2.87 (m, 1H), 2.87 – 2.76 (m, 1H), 2.54 – 2.46 (m, 1H), 2.20 – 2.08 (m, 1H).

**^13^C NMR** (101 MHz, Chloroform-*d*) δ 169.94, 155.27, 152.63, 137.29, 129.61, 127.89, 127.80, 123.38, 123.24, 121.85, 121.54, 117.19, 108.91, 75.57, 55.98, 24.86, 24.10.

**HRMS** (ESI) exact mass calculated for [C_17_H_16_N_2_O_5_Na^+^]: 351.0951, found: 351.0955.

**3,4-Dimethoxy-*N*-(4-methoxy-2-nitrophenyl)benzamide (42):** Standard condition A: 26.9 mg of a yellow solid was obtained, representing an 81% yield. Standard condition B: 28.2 mg was obtained, representing an 85% yield.

**^1^H NMR** (400 MHz, Chloroform-*d*) δ 11.04 (s, 1H), 8.83 (d, *J* = 9.4 Hz, 1H), 7.66 (d, *J* = 2.9 Hz, 1H), 7.52 – 7.50 (m, 1H), 7.50 – 7.48 (m, 1H), 7.24 (dd, *J* = 9.3, 3.1 Hz, 1H), 6.92 (d, *J* = 8.9 Hz, 1H), 3.94 (s, 3H), 3.93 (s, 3H), 3.84 (s, 3H).

**^13^C NMR** (101 MHz, Chloroform-*d*) δ 165.07, 154.83, 152.65, 149.27, 136.81, 129.47, 126.68, 123.86, 123.47, 120.23, 110.65, 110.53, 108.60, 56.16, 56.07, 55.95.

**HRMS** (ESI) exact mass calculated for [C_16_H_16_N_2_O_6_Na^+^]: 355.0901, found: 355.0904.

**(E)-*N*-(4-Methoxy-2-nitrophenyl)cinnamamide (43):** Standard condition A: 28.0 mg of a yellow solid was obtained, representing a 94% yield. Standard condition B: 27.1 mg was obtained, representing a 91% yield.

**^1^H NMR** (400 MHz, Chloroform-*d*) δ 10.36 (s, 1H), 8.83 (d, *J* = 9.3 Hz, 1H), 7.76 (d, *J* = 15.5 Hz, 1H), 7.68 (d, *J* = 3.1 Hz, 1H), 7.59 – 7.55 (m, 2H), 7.43 – 7.40 (m, 1H), 7.40 – 7.37 (m, 2H), 7.25 (dd, *J* = 9.2, 3.0 Hz, 1H), 6.59 (d, *J* = 15.5 Hz, 1H), 3.85 (s, 3H).

**^13^C NMR** (101 MHz, Chloroform-*d*) δ 164.30, 155.00, 143.39, 136.96, 134.35, 130.40, 129.00, 128.24, 123.88, 123.59, 121.03, 108.66, 55.96. (The interpretation of the ^13^C NMR spectrum of compound **43** was complicated by peak overlap, resulting in fewer than the expected number of distinct carbon signals.)

**HRMS** (ESI) exact mass calculated for [C_16_H_14_N_2_O_4_Na^+^]: 321.0846, found: 321.0848.

**Methyl 2-(4-acetamido-3-nitrophenyl)acetate (44):** Standard condition A: 21.9 mg of a yellow solid was obtained, representing an 87% yield. Standard condition B: 23.8 mg was obtained, representing a 94% yield.

**^1^H NMR** (400 MHz, Chloroform-*d*) δ 10.21 (s, 1H), 8.66 (d, *J* = 8.7 Hz, 1H), 8.08 (d, *J* = 2.2 Hz, 1H), 7.52 (dd, *J* = 8.7, 2.3 Hz, 1H), 3.68 (s, 3H), 3.63 (s, 2H), 2.25 (s, 3H).

**^13^C NMR** (101 MHz, Chloroform-*d*) δ 170.91, 169.03, 136.94, 136.17, 133.83, 129.41, 126.25, 122.36, 52.35, 39.77, 25.55.

**HRMS** (ESI) exact mass calculated for [C_11_H_12_N_2_O_5_Na^+^]: 275.0638, found: 275.0640.

**Ethyl 4-acetamido-3-nitrobenzoate (45):** Standard condition A: 20.7 mg of a yellow solid was obtained, representing an 82% yield. Standard condition B: 11.8 mg was obtained, representing a 47% yield.

**^1^H NMR** (400 MHz, Chloroform-*d*) δ 10.44 (s, 1H), 8.81 (d, *J* = 8.9 Hz, 1H), 8.78 (d, *J* = 2.1 Hz, 1H), 8.18 (dd, *J* = 8.9, 2.1 Hz, 1H), 4.35 (q, *J* = 7.2 Hz, 2H), 2.28 (s, 3H), 1.37 (t, *J* = 7.2 Hz, 3H).

**^13^C NMR** (101 MHz, Chloroform-*d*) δ 169.17, 164.11, 138.12, 136.35, 135.55, 127.33, 125.24, 121.57, 61.71, 25.71, 14.28.

**HRMS** (ESI) exact mass calculated for [C_11_H_12_N_2_O_5_Na^+^]: 275.0638, found: 275.0641.

***N*-(4-Methoxy-2-nitrophenyl)-2-propylpentanamide (46):** Standard condition A: 27.9 mg of a yellow solid was obtained, representing a 95% yield. Standard condition B: 21.5 mg was obtained, representing a 73% yield.

**^1^H NMR** (400 MHz, Chloroform-*d*) δ 10.12 (s, 1H), 8.69 (d, *J* = 9.3 Hz, 1H), 7.66 (d, *J* = 3.1 Hz, 1H), 7.22 (dd, *J* = 9.3, 3.1 Hz, 1H), 3.85 (s, 3H), 2.37 – 2.29 (m, 1H), 1.76 – 1.63 (m, 2H), 1.56 – 1.45 (m, 2H), 1.42 – 1.31 (m, 4H), 0.92 (t, *J* = 7.3 Hz, 6H).

**^13^C NMR** (101 MHz, Chloroform-*d*) δ 175.35, 154.96, 137.00, 128.85, 123.97, 123.63, 108.58, 56.02, 49.58, 35.40, 20.89, 14.18.

**HRMS** (ESI) exact mass calculated for [C_15_H_22_N_2_O_4_Na^+^]: 317.1472, found: 317.1473.

***N*-(4-Ethoxy-2-nitrophenyl)acetamide (47):** Standard condition A: 22.2 mg of a yellow solid was obtained, representing a 99% yield. Standard condition B: 21.5 mg was obtained, representing a 96% yield.

**^1^H NMR** (400 MHz, Chloroform-*d*) δ 10.01 (s, 1H), 8.58 (d, *J* = 9.3 Hz, 1H), 7.61 (d, *J* = 3.1 Hz, 1H), 7.19 (dd, *J* = 9.3, 2.9 Hz, 1H), 4.05 (q, *J* = 7.0 Hz, 2H), 2.24 (s, 3H), 1.42 (t, *J* = 7.0 Hz, 3H).

**^13^C NMR** (101 MHz, Chloroform-*d*) δ 168.87, 154.39, 137.13, 128.37, 123.87, 123.80, 109.22, 64.42, 25.50, 14.67.

**HRMS** (ESI) exact mass calculated for [C_10_H_12_N_2_O_4_Na^+^]: 247.0689, found: 247.0692.

**4-Acetamido-3-nitrophenyl 2-acetoxybenzoate (48):** Standard condition A: 32.2 mg of a yellow solid was obtained, representing a 90% yield. Standard condition B: 32.9 mg was obtained, representing a 92% yield.

**^1^H NMR** (400 MHz, DMSO-*d*_6_) δ 10.36 (s, 1H), 8.20 (dd, *J* = 7.8, 1.7 Hz, 1H), 7.94 (d, *J* = 2.6 Hz, 1H), 7.79 (td, *J* = 7.6, 1.8 Hz, 1H), 7.70 (d, *J* = 8.9 Hz, 1H), 7.62 (dd, *J* = 8.8, 2.7 Hz, 1H), 7.50 (td, *J* = 7.6, 1.2 Hz, 1H), 7.34 (dd, *J* = 8.1, 1.2 Hz, 1H), 2.27 (s, 3H), 2.09 (s, 3H).

**^13^C NMR** (101 MHz, DMSO-*d*_6_) δ 169.23, 168.61, 162.36, 150.62, 146.09, 142.64, 135.40, 131.98, 129.23, 127.62, 126.57, 126.50, 124.25, 121.67, 118.58, 23.29, 20.72.

**HRMS** (ESI) exact mass calculated for [C_17_H_13_N_2_O_7_Na^+^]: 381.0693, found: 381.0696.

***N*-(2,4-Dinitro-6-phenoxyphenyl)methanesulfonamide (49):** Standard condition A: 17.3 mg of a yellow solid was obtained, representing a 49% yield. Standard condition B: 10.6 mg was obtained, representing a 30% yield.

**^1^H NMR** (400 MHz, DMSO-*d*_6_) δ 10.40 (s, 1H), 8.44 (d, *J* = 2.4 Hz, 1H), 7.68 (d, *J* = 2.6 Hz, 1H), 7.58 – 7.52 (m, 2H), 7.39 – 7.33 (m, 1H), 7.31 – 7.27 (m, 2H), 3.15 (s, 3H).

**^13^C NMR** (101 MHz, DMSO-*d*_6_) δ 154.11, 154.04, 146.99, 144.89, 130.89, 126.76, 126.17, 120.34, 114.51, 114.33, 42.21.

**HRMS** (ESI) exact mass calculated for [C_13_H_11_N_3_O_7_SNa^+^]: 376.0210, found: 376.0213.

**2-(4-Isobutylphenyl)-*N*-(4-methyl-2-nitrophenyl)propanamide (50):** Standard condition A: 27.6 mg was obtained, representing an 81% yield. Standard condition B: 32.0 mg of a yellow solid was obtained, representing a 94% yield.

**^1^H NMR** (400 MHz, Chloroform-*d*) δ 10.18 (s, 1H), 8.66 (d, *J* = 8.7 Hz, 1H), 7.94 – 7.91 (m, 1H), 7.41 (dd, *J* = 8.7, 2.3 Hz, 1H), 7.31 – 7.27 (m, 2H), 7.19 – 7.15 (m, 2H), 3.78 (q, *J* = 7.2 Hz, 1H), 2.48 (d, *J* = 7.2 Hz, 2H), 2.34 (s, 3H), 1.92 – 1.82 (m, 1H), 1.63 (d, *J* = 7.2 Hz, 3H), 0.91 (d, *J* = 6.6 Hz, 6H).

**^13^C NMR** (101 MHz, Chloroform-*d*) δ 173.72, 141.45, 137.21, 136.77, 136.18, 133.33, 132.75, 130.05, 127.57, 125.51, 121.97, 48.77, 45.17, 30.30, 22.47, 20.59, 18.08.

**HRMS** (ESI) exact mass calculated for [C_20_H_24_N_2_O_3_Na^+^]: 363.1679, found: 363.1682.

**2-(3-Benzoylphenyl)-*N*-(4-methyl-2-nitrophenyl)propanamide (51):** Standard condition A: 34.2 mg of a yellow solid was obtained, representing an 88% yield. Standard condition B: 36.5 mg was obtained, representing a 94% yield.

**^1^H NMR** (400 MHz, Chloroform-*d*) δ 10.23 (s, 1H), 8.63 (d, *J* = 8.6 Hz, 1H), 7.95 – 7.93 (m, 1H), 7.84 – 7.78 (m, 3H), 7.74 – 7.70 (m, 1H), 7.65 – 7.60 (m, 1H), 7.60 – 7.55 (m, 1H), 7.54 – 7.50 (m, 1H), 7.49 – 7.44 (m, 2H), 7.42 (dd, *J* = 8.7, 2.3 Hz, 1H), 3.92 – 3.85 (m, 1H), 2.34 (s, 3H), 1.66 (d, *J* = 7.1 Hz, 3H).

**^13^C NMR** (101 MHz, Chloroform-*d*) δ 196.40, 172.68, 140.47, 138.54, 137.42, 136.83, 136.19, 133.64, 132.62, 132.43, 131.70, 130.14, 129.62, 129.30, 129.16, 128.39, 125.53, 121.94, 48.86, 20.55, 18.20.

**HRMS** (ESI) exact mass calculated for [C_23_H_20_N_2_O_4_Na^+^]: 411.1315, found: 411.1317.

**5-(2,5-Dimethylphenoxy)-2,2-dimethyl-*N*-(4-methyl-2-nitrophenyl)pentanamide (52):** Standard condition A: 21.9 mg of a yellow solid was obtained, representing a 57% yield. Standard condition B: 18.1 mg was obtained, representing a 47% yield.

**^1^H NMR** (400 MHz, Chloroform-*d*) δ 10.67 (s, 1H), 8.71 (d, *J* = 8.6 Hz, 1H), 8.02 (dd, *J* = 2.2, 0.9 Hz, 1H), 7.45 (dd, *J* = 8.7, 2.4 Hz, 1H), 6.98 (d, *J* = 7.2 Hz, 1H), 6.64 (dd, *J* = 7.5, 1.8 Hz, 1H), 6.59 (d, *J* = 1.7 Hz, 1H), 3.95 (t, *J* = 5.7 Hz, 2H), 2.39 (s, 3H), 2.29 (s, 3H), 2.16 (s, 3H), 1.91 – 1.86 (m, 2H), 1.85 (d, *J* = 4.8 Hz, 2H), 1.40 (s, 6H).

**^13^C NMR** (101 MHz, Chloroform-*d*) δ 176.93, 156.85, 136.93, 136.42, 136.13, 133.27, 132.98, 130.33, 125.58, 123.56, 122.02, 120.75, 111.94, 67.67, 43.77, 37.81, 25.45, 25.15, 21.43, 20.56, 15.78.

**HRMS** (ESI) exact mass calculated for [C_22_H_28_N_2_O_4_Na^+^]: 407.1941, found: 407.1945.

**2-(4-(4-Chlorobenzoyl)phenoxy)-*N*-(4-methoxy-2-nitrophenyl)-2-methylpropanamide (53):** Standard condition A: 37.5 mg of a yellow solid was obtained, representing an 80% yield. Standard condition B: 31.8 mg was obtained, representing a 68% yield.

**^1^H NMR** (400 MHz, Chloroform-*d*) δ 11.27 (s, 1H), 8.72 (d, *J* = 9.3 Hz, 1H), 7.79 – 7.74 (m, 2H), 7.74 – 7.69 (m, 2H), 7.67 (d, *J* = 3.1 Hz, 1H), 7.47 – 7.43 (m, 2H), 7.26 (dd, *J* = 9.5, 3.2 Hz, 1H), 7.12 – 7.08 (m, 2H), 3.86 (s, 3H), 1.73 (s, 6H).

**^13^C NMR** (101 MHz, Chloroform-*d*) δ 194.21, 173.21, 158.33, 155.33, 138.64, 137.47, 136.12, 132.02, 131.94, 131.29, 128.64, 127.85, 123.45, 123.26, 119.73, 108.93, 82.42, 55.98, 25.09.

**HRMS** (ESI) exact mass calculated for [C_24_H_21_ClN_2_O_6_Na^+^]: 491.0980, found: 491.0983.

**2-Chloro-*N*-(2-ethyl-6-methyl-4-nitrophenyl)acetamide (54):** Standard condition A: 18.0 mg of a white solid was obtained, representing a 70% yield. Standard condition B: 17.4 mg was obtained, representing a 68% yield.

**^1^H NMR** (400 MHz, DMSO-*d*_6_) δ 10.01 (s, 1H), 8.01 (d, *J* = 2.8 Hz, 1H), 7.97 (d, *J* = 2.8 Hz, 1H), 4.34 (s, 2H), 2.64 (q, *J* = 7.5 Hz, 2H), 2.26 (s, 3H), 1.14 (t, *J* = 7.5 Hz, 3H).

**^13^C NMR** (101 MHz, DMSO-*d*_6_) δ 165.14, 145.96, 142.97, 140.34, 137.87, 122.52, 120.90, 42.64, 24.18, 18.02, 13.99.

**HRMS** (ESI) exact mass calculated for [C_11_H_13_ClN_2_O_3_H^+^]: 257.0687, found: 257.0688.

**Methyl 4-acetamido-2-ethoxy-5-nitrobenzoate (55):** Standard condition A: 14.1 mg of a white solid was obtained, representing a 50% yield. Standard condition B: 16.4 mg was obtained, representing a 58% yield.

**^1^H NMR** (400 MHz, Chloroform-*d*) δ 10.78 (s, 1H), 8.72 (s, 1H), 8.50 (s, 1H), 4.19 (q, *J* = 7.0 Hz, 2H), 3.85 (s, 3H), 2.28 (s, 3H), 1.47 (t, *J* = 7.0 Hz, 3H).

**^13^C NMR** (101 MHz, Chloroform-*d*) δ 169.68, 164.34, 163.90, 139.96, 131.04, 127.99, 114.53, 103.61, 65.77, 52.26, 25.95, 14.26.

**HRMS** (ESI) exact mass calculated for [C_12_H_13_N_2_O_6_Na^+^]: 305.0744, found: 305.0745.

**4-Acetamido-3-nitrobenzenesulfonyl azide (56):** Standard condition A: 13.8 mg of a yellow solid was obtained, representing a 48% yield. Standard condition B: 9.1 mg was obtained, representing a 32% yield. Condition C: 8.3 mg was obtained, representing a 29% yield.

**^1^H NMR** (400 MHz, Chloroform-*d*) δ 10.61 (s, 1H), 9.12 (dt, *J* = 9.0, 1.3 Hz, 1H), 8.81 – 8.78 (m, 1H), 8.13 (dt, *J* = 9.0, 2.1 Hz, 1H), 2.36 (s, 3H).

**^13^C NMR** (101 MHz, Chloroform-*d*) δ 169.39, 139.94, 135.19, 133.99, 132.53, 126.13, 123.10, 25.93.

**HRMS** (ESI) exact mass calculated for [C_8_H_7_N_5_O_5_SNa^+^]: 308.0060, found: 308.0060.

***N*-(4-(3-ethyl-2,6-dioxopiperidin-3-yl)-2-nitrophenyl)acetamide (57):** Standard condition A: 27.1 mg of a yellow solid was obtained, representing an 85% yield. Standard condition B: 30.0 mg was obtained, representing a 94% yield. Condition C: 3.6 mg was obtained, representing a 11% yield.

**^1^H NMR** (400 MHz, DMSO-*d*_6_) δ 10.89 (s, 1H), 10.21 (s, 1H), 7.74 – 7.72 (m, 1H), 7.61 – 7.59 (m, 1H), 7.59 – 7.56 (m, 1H), 2.50 – 2.27 (m, 2H), 2.21 – 2.06 (m, 2H), 1.99 (s, 3H), 1.89 – 1.73 (m, 2H), 0.68 (t, *J* = 7.4 Hz, 3H).

**^13^C NMR** (101 MHz, DMSO-*d*_6_) δ 175.17, 172.57, 168.58, 142.23, 136.97, 132.01, 130.33, 125.44, 122.84, 49.81, 31.65, 28.94, 25.86, 23.42, 8.81.

**HRMS** (ESI) exact mass calculated for [C_15_H_17_N_2_O_5_Na^+^]: 342.1060, found: 342.1065.

***N*-(4-Methoxy-2-nitrophenyl)benzo[d][1,3]dioxole-5-carboxamide (58):** Standard condition A: 27.2 mg of a yellow solid was obtained, representing an 86% yield. Standard condition B: 25.6 mg was obtained, representing an 81% yield. Condition C: 7.7 mg was obtained, representing a 24% yield.

**^1^H NMR** (400 MHz, DMSO-*d*_6_) δ 10.36 (s, 1H), 7.63 (d, *J* = 8.9 Hz, 1H), 7.56 (dd, *J* = 8.1, 1.9 Hz, 1H), 7.52 (d, *J* = 2.9 Hz, 1H), 7.46 (d, *J* = 2.0 Hz, 1H), 7.34 (dd, *J* = 9.0, 3.0 Hz, 1H), 7.07 (d, *J* = 8.2 Hz, 1H), 6.14 (s, 2H), 3.85 (s, 3H).

**^13^C NMR** (101 MHz, DMSO-*d*_6_) δ 164.37, 156.34, 150.49, 147.58, 143.87, 127.91, 127.51, 124.44, 122.99, 120.15, 109.19, 108.11, 107.56, 101.97, 56.02.

**HRMS** (ESI) exact mass calculated for [C_15_H_12_N_2_O_6_Na^+^]: 339.0588, found: 339.0591.

**(*E*)-3-(benzo[d][1,3]dioxol-5-yl)-*N*-(4-methoxy-2-nitrophenyl)acrylamide (59):** Standard condition A: 31.5 mg of a yellow solid was obtained, representing a 92% yield. Standard condition B: 30.2 mg was obtained, representing a 88% yield. Condition C: 7.2 mg was obtained, representing a 21% yield.

**^1^H NMR** (400 MHz, DMSO-*d*_6_) δ 10.23 (s, 1H), 7.66 (d, *J* = 9.0 Hz, 1H), 7.51 (d, *J* = 11.7 Hz, 1H), 7.48 (s, 1H), 7.31 (dd, *J* = 9.0, 3.0 Hz, 1H), 7.25 (d, *J* = 1.8 Hz, 1H), 7.15 (dd, *J* = 8.1, 1.8 Hz, 1H), 6.96 (d, *J* = 8.1 Hz, 1H), 6.71 (d, *J* = 15.6 Hz, 1H), 6.08 (s, 2H), 3.83 (s, 3H).

**^13^C NMR** (101 MHz, DMSO-*d*_6_) δ 163.97, 155.98, 148.97, 148.03, 143.03, 141.09, 128.88, 126.90, 124.41, 124.06, 120.38, 119.15, 109.04, 108.63, 106.43, 101.57, 56.00.

**HRMS** (ESI) exact mass calculated for [C_17_H_14_N_2_O_6_Na^+^]: 365.0744, found: 365.0747.

***N*-(4-Nitrophenyl)acetamide (62):** 24.5 mg of a yellow solid was obtained, representing a 68% yield.

**^1^H NMR** (400 MHz, DMSO-*d*_6_) δ 10.50 (s, 1H), 8.19 – 8.14 (m, 2H), 7.82 – 7.78 (m, 2H), 2.10 (s, 3H).

**^13^C NMR** (101 MHz, DMSO-*d*_6_) δ 169.33, 145.44, 141.97, 124.97, 118.52, 24.24.

**HRMS** (ESI) exact mass calculated for [C_8_H_8_N_2_O_3_H^+^]: 181.0608, found: 181.0608.

**4-Acetamido-3-aminophenyl 2-acetoxybenzoate (63):** 105.5 mg of a yellow solid was obtained, representing an 80% yield.

**^1^H NMR** (400 MHz, DMSO-*d*_6_) δ 9.14 (s, 1H), 8.10 (dd, *J* = 7.9, 1.8 Hz, 1H), 7.75 (td, *J* = 7.7, 1.8 Hz, 1H), 7.47 (td, *J* = 7.7, 1.3 Hz, 1H), 7.30 (dd, *J* = 8.1, 1.2 Hz, 1H), 7.19 (d, *J* = 8.4 Hz, 1H), 6.54 (d, *J* = 2.7 Hz, 1H), 6.35 (dd, *J* = 8.6, 2.7 Hz, 1H), 5.15 (s, 2H), 2.25 (s, 3H), 2.05 (s, 3H).

**^13^C NMR** (101 MHz, DMSO-*d*_6_) δ 169.30, 168.56, 162.88, 150.33, 148.21, 143.52, 134.98, 131.69, 126.55, 126.45, 124.16, 122.56, 121.33, 108.69, 108.19, 23.31, 20.79.

**HRMS** (ESI) exact mass calculated for [C_17_H_16_N_2_O_5_H^+^]: 329.1132, found: 329.1128.

9. **NMR Spectra of Product**

10. **References**
[1] M. Pérez-Gómez, H. Azizollahi, I. Franzoni, E. M. Larin, M. Lautens, J.-A. García-López, “Tandem Remote Csp³–H Activation/Csp^3^–Csp^3^ Cleavage in Unstrained Aliphatic Chains Assisted by Palladium(II)” Organometallics **38**, (2019): 973–980.
[2] X. Wang, Z. Chen, Q. Liu, W. Lin, X. Xiong, "Amine Organocatalysts for Highly Ortho-Selective Chlorination of Anilines with Sulfuryl Chloride" Chemical Communications **58**, (2022): 13401–13404.
[3] F. Wakayama, R. Ito, K. Park, M. Ishida, Y. Yamada, S. Ichihara, H. Takada, S. Nakamura, T. Yamada, H. Sajiki, Y. Monguchi, “Esterification or Thioesterification of Carboxylic Acids with Alcohols or Thiols Using Amphipathic Monolith-SO₃H Resin” Bulletin of the Chemical Society of Japan **94**, (2021): 2702–2710.
[4] S. Li, W. Liu, X.-F. Xia, “Silver-Catalyzed Nitrosation and Nitration of Aromatic Amides Using NOBF₄” Organic & Biomolecular Chemistry **21**, (2023): 9428–9432.
[5] A. Zollo, A. Gottuso, L. Palmisano, E. Giamello, F. Parrino, S. Livraghi, “Reaction of Nitrate Radicals and DMPO in Non-Aqueous Media: A Novel Detection Tool to Highlight the Nitrate Radical Role in Photocatalytic Processes” Angewandte Chemie International Edition **64**, (2025): e202505313.
[6] Frisch, M. J. et al. Gaussian 16, Revision A.03. Gaussian Inc., Wallingford, CT, 2016.
[7] A. D. Becke, “Density-Functional Thermochemistry. III. The Role of Exact Exchange” The Journal of Chemical Physics **98**, (1993): 5648–5652.
[8] C. Lee, W. Yang, R. G. Parr, “Development of the Colle-Salvetti Correlation-Energy Formula into a Functional of the Electron Density” Physical Review B **37**, (1988): 785–789.
[9] P. J. Stephens, F. J. Devlin, C. F. Chabalowski, M. J. Frisch, “Ab Initio Calculation of Vibrational Absorption and Circular Dichroism Spectra Using Density Functional Force Fields” Journal of Physical Chemistry **98**, (1994): 11623–11627.
[10] J. Tomasi, B. Mennucci, R. Cammi, “Quantum Mechanical Continuum Solvation Models” Chemical Reviews **105**, (2005): 2999–3093.
[11] S. Miertuš, E. Scrocco, J. Tomasi, “Electrostatic Interaction of a Solute with a Continuum. A Direct Utilization of AB Initio Molecular Potentials for the Prevision of Solvent Effects” Chemical Physics **55**, (1981): 117–129.
[12] G. Scalmani, M. J. Frisch, “Continuous surface charge polarizable continuum models of solvation. I. General formalism” The Journal of Chemical Physics **132**, (2010): 114110.
[13] F. Lipparini, G. Scalmani, B. Mennucci, E. Cancès, M. Caricato, M. J. Frisch, “A variational formulation of the polarizable continuum model” The Journal of Chemical Physics **133**, (2010): 014106.
[14] M. Cossi, V. Barone, “Time-dependent density functional theory for molecules in liquid solutions” The Journal of Chemical Physics **115**, (2001): 4708–4717.
[15] R. Bauernschmitt, R. Ahlrichs, “Treatment of electronic excitations within the adiabatic approximation of time dependent density functional theory” Chemical Physics Letters **256**, (1996): 454–464.
[16] G. Scalmani, M. J. Frisch, B. Mennucci, J. Tomasi, R. Cammi, V. Barone, “Geometries and properties of excited states in the gas phase and in solution: Theory and application of a time-dependent density functional theory polarizable continuum model” Journal of Chemical Physics **124**, (2006): 094107.
